# Optimal Runge approximation for nonlocal wave equations and unique determination of polyhomogeneous nonlinearities

**DOI:** 10.1007/s00526-025-03124-0

**Published:** 2025-10-10

**Authors:** Yi-Hsuan Lin, Teemu Tyni, Philipp Zimmermann

**Affiliations:** 1 Department of Applied Mathematics, National Yang Ming Chiao Tung University, Hsinchu, Taiwan; 2 Fakultät für Mathematik, University of Duisburg-Essen, Essen, Germany; 3 Research Unit of Applied and Computational Mathematics, University of Oulu, Oulu, Finland; 4 Departament de Matemàtiques i Informàtica, Universitat de Barcelona, Barcelona, Spain

**Keywords:** Primary 35R30, Secondary 26A33, 42B37

## Abstract

The main purpose of this article is to establish the Runge-type approximation in $L^2(0,T;\widetilde{H}^s(\Omega ))$ for solutions of linear nonlocal wave equations. To achieve this, we extend the theory of very weak solutions for classical wave equations to our nonlocal framework. This strengthened Runge approximation property allows us to extend the existing uniqueness results for Calderón problems of linear and nonlinear nonlocal wave equations in our earlier works. Furthermore, we prove unique determination results for the Calderón problem of nonlocal wave equations with polyhomogeneous nonlinearities.

## Introduction

In recent years, inverse problems for nonlocal partial differential equations (PDEs) have been extensively studied in the literature. The first work in this field is [18], in which the authors considered the so-called *Calderón problem* for the *fractional Schrödinger equation*

1.1 $$\begin{aligned} ((-\Delta )^s+q)u=0\text { in }\Omega , \end{aligned}$$

where $\Omega \subset {{\mathbb {R}}}^n$ is a bounded domain. Here $(-\Delta )^s$ denotes the *fractional Laplacian* for $0<s<1$, and $q\in L^{\infty }(\Omega )$ is a bounded potential. In this problem, one asks whether it is possible to uniquely recover the potential *q* from the *Dirichlet-to-Neumann (DN) map*

1.2 $$\begin{aligned} \Lambda _q f{:}= (-\Delta )^s u_f\big |_{\Omega _e}, \end{aligned}$$

where $\Omega _e={{\mathbb {R}}}^n\setminus \overline{\Omega }$ denotes the exterior of $\Omega$, $f:\Omega _e\rightarrow {{\mathbb {R}}}$ is a given Dirichlet data, and $u_f:{{\mathbb {R}}}^n\rightarrow {{\mathbb {R}}}$ is the unique solution of (1.1) with $\left. u_f\right| _{\Omega _e}=f$. In [18], the authors found that the fractional Laplacian satisfies the *unique continuation principle (UCP)* that asserts:

**(UCP)**. *Let* $s\in {{\mathbb {R}}}_+\setminus {{\mathbb {N}}}$, $t\in {{\mathbb {R}}}$ *and* $V\subset {{\mathbb {R}}}^n$ *be an open set. If* $u\in H^t({{\mathbb {R}}}^n)$ *satisfies*

$$ u=(-\Delta )^s u=0\text { in }V \text { then } u\equiv 0 \text { in } {{\mathbb {R}}}^n. $$

In [18] the UCP has been shown for the range $0<s<1$ and via iteration by the Laplacian the UCP extends to the range $s\in {{\mathbb {R}}}_+\setminus {{\mathbb {N}}}$. Furthermore, they observe that the fractional Laplacian has, as a consequence of a duality argument (Hahn–Banach theorem) and the UCP, the so-called *Runge approximation property*. For any open set $W\subset \Omega _e$, this property can be phrased in two alternative ways:

(i) The *Runge set*
  $$ \mathcal {R}_W{:}=\big \{ u_{f}\big |_{\Omega } \,;\, f\in C^\infty _c (W) \big \} $$
  is dense in $L^2(\Omega )$, where $u_f$ is the unique solution to (1.2) with exterior value $f\in C_c^{\infty }(W)$ (cf. [18]).
(ii) The *Runge set*
  $$ \mathscr {R}_W{:}=\big \{ u_{f}-f \,;\, f\in C^\infty _c (W) \big \} $$
  is dense in $\widetilde{H}^s(\Omega )$ (cf. [49]).

Together with a suitable integral identity, the result (i) allowed the authors of [18] to uniquely recover bounded potentials, whereas the property (ii) was used in [49] to recover certain singular potentials.

The above strategy to establish uniqueness for Calderón-type inverse problems of elliptic or parabolic nonlocal equations has lately been investigated in several research articles, such as [8–11, 14–17, 27, 28, 31, 32, 38, 38, 42, 43, 46, 47, 50, 51]. Some articles of this list consider the detection of linear perturbations as in the above fractional Schrödinger equation (1.1), while others allowed nonlinear perturbations, or even studied the identification of leading order coefficients in the main nonlocal term in the considered PDE. We also point out a recent monograph [33] for a comprehensive introduction to this research field.

### The mathematical model and main results

In this article, we study Calderón-type inverse problems for linear and nonlinear *nonlocal wave equations (NWEQs)* formulated generically as

1.3 $$\begin{aligned} {\left\{ \begin{array}{ll} \partial _t^2u +(-\Delta )^su+f(x,u)=0 & \text { in }\Omega _T,\\ u=\varphi & \text { in }(\Omega _e)_T,\\ u(0)= \partial _t u(0)=0 & \text { in }{{\mathbb {R}}}^n, \end{array}\right. } \end{aligned}$$

where $f:\Omega \times {{\mathbb {R}}}\rightarrow {{\mathbb {R}}}$ is a suitable nonlinearity. Here we use the notation

$$\begin{aligned} A_t {:}= A\times (0,t), \text { for any }A\subset {{\mathbb {R}}}^n\text { and }t>0. \end{aligned}$$

Let us note that nonlocal wave equations such as (1.3) arise in a special case of *peridynamics* — the theory of studying dynamics of materials with discontinuities such as fractures (see [52]). Meanwhile, for the well-posedness of (1.3), we need the compatibility condition to match the initial value such that the exterior data satisfies $\varphi (x,0)=0$ in $\Omega _e$.

By [43, Theorem 3.1 and 3.6] the equation (1.3) is well-posed for regular exterior values $\varphi :\Omega _e\rightarrow {{\mathbb {R}}}$, when $f(x,\tau )=q(x)\tau$ with $q\in L^p(\Omega )$, where $1\le p\le \infty$ satisfies

1.4 $$\begin{aligned} {\left\{ \begin{array}{ll} \frac{n}{s}\le p\le \infty , & \, \text {if }\, 2s< n,\\ 2<p\le \infty , & \, \text {if }\, 2s= n,\\ 2\le p\le \infty , & \, \text {if }\, 2s\ge n, \end{array}\right. } \end{aligned}$$

or *f* satisfies the following assumption:

#### Assumption 1

We say that a Carathéodory function $f:\Omega \times {{\mathbb {R}}}\rightarrow {{\mathbb {R}}}$ is a *weak nonlinearity* if it satisfies the following conditions:

(i) *f* has partial derivative $\partial _{\tau }f$, which is a Carathéodory function, and there exists $a\in L^p(\Omega )$ such that
  $$\begin{aligned} \left| \partial _\tau f(x,\tau )\right| \lesssim a(x)+|\tau |^r \end{aligned}$$
  for all $\tau \in {{\mathbb {R}}}$ and a.e. $x\in \Omega$. Here the exponents *p* and *r* satisfy the restrictions (1.4) and
  1.5 $$\begin{aligned} {\left\{ \begin{array}{ll} 0\le r<\infty , & \, \text {if }\, 2s\ge n,\\ 0\le r\le \frac{2s}{n-2s}, & \, \text {if }\, 2s< n, \end{array}\right. } \end{aligned}$$
  respectively. Moreover, *f* fulfills the integrability condition $f(\cdot ,0)\in L^2(\Omega )$.
(ii) There is a constant $C_1>0$ such that the function $F:\Omega \times {{\mathbb {R}}}\rightarrow {{\mathbb {R}}}$ defined via
  $$ F(x,\tau )=\int _0^\tau f(x,\rho )\,d\rho $$
  satisfies $F(x,\tau )\ge -C_1$ for all $\tau \in {{\mathbb {R}}}$ and $x\in \Omega$.

Observe that given a function $0\le q\in L^\infty (\Omega )$, an example of a nonlinearity *f*, which satisfies the conditions in Assumption 1, is a fractional power type nonlinearity $f(x,\tau )=q(x)|\tau |^r\tau$ for $r\ge 0$ with *r* satisfying (1.5). We refer readers to [43, Section 3] for more details.

Assuming the well-posedness of (1.3) for suitable nonlinearities *f*, as a generalization of the Calderón problem for the fractional Schrödinger equation, we aim to determine the nonlinearity $f(x,\tau )$ from the DN map $\Lambda _f$ related to (1.3), which can be formally defined by

$$\begin{aligned} \Lambda _{f}\varphi {:}= (-\Delta )^s u_\varphi \big |_{(\Omega _e)_T}, \end{aligned}$$

where $u_\varphi :{{\mathbb {R}}}^n_T\rightarrow {{\mathbb {R}}}$ denotes the unique solution to (1.3) (cf. eq. (1.2)). Next, let us make a few remarks on this inverse problem.

- *Linear perturbations:* In [27], the uniqueness of this inverse problem has been established in the case $f(x,u)=q(x)u$ with $q\in L^{\infty }(\Omega )$. In [43, Corollary 1.4], it has been shown that uniqueness still holds if $0\le q\in L^p(\Omega )$ with *p* satisfying (1.4).
- *Semilinear perturbations:* In [43, Theorem 1.1], we showed that uniqueness holds even when
  (A) *f* is a weak nonlinearity (see Assumption 1) with *F*, *r* satisfying
    1. $$F\ge 0$$,
    2. $$0<r\le 1$$
  (B) and *f* is $(r+1)-$homogeneous.

All of these results rely on a $L^2(\Omega _T)$ Runge approximation property of linear nonlocal wave equations (cf. (i)):

#### Proposition 1.1

(Runge approximation, [43, Proposition 4.1]) Let $\Omega \subset {{\mathbb {R}}}^n$ be a bounded Lipschitz domain, $W\subset \Omega _e$ an arbitrary open set, $s>0$ a non-integer and $T>0$. Suppose that $q\in L^p(\Omega )$ is nonnegative^1^, where *p* is given by (1.4). Consider the *Runge set*

$$\begin{aligned} \mathcal {R}_W{:}=\big \{ u_{\varphi }\big |_{\Omega _T} \,;\, \varphi \in C^\infty _c (W_T) \big \}, \end{aligned}$$

where $u_\varphi \in C([0,T];H^s({{\mathbb {R}}}^n))\cap C^1([0,T]; L^2({{\mathbb {R}}}^n))$ is the unique solution to

1.6 $$\begin{aligned} {\left\{ \begin{array}{ll} \partial _t^2u +(-\Delta )^su+qu = 0 & \text { in }\Omega _T,\\ u=\varphi & \text { in }(\Omega _e)_T,\\ u(0)= \partial _t u(0)=0 & \text { in }\Omega . \end{array}\right. } \end{aligned}$$

Then $\mathcal {R}_W$ is dense in $L^2(\Omega _T)$.

The main goal of this article is to answer the question raised in [54, p. 3]:

#### Question 1

Let us adopt all the notation of Proposition 1.1. Is the Runge set

$$\begin{aligned} \mathscr {R}_W{:}=\left\{ u_{\varphi }-\varphi \,;\, \varphi \in C^\infty _c (W_T) \right\} \end{aligned}$$

dense in $$L^2(0,T;\widetilde{H}^s(\Omega ))$$?

An affirmative answer to this question will be established by extending the theory of very weak solutions to linear nonlocal wave equations (Section 3) and it forms the core contribution of our article as it provides an essential tool for studying the subsequent inverse problems. It reads as follows:

#### Theorem 1.2

(Optimal Runge approximation) Let $\Omega \subset {{\mathbb {R}}}^n$ be a bounded Lipschitz domain, $W\subset \Omega _e$ an arbitrary open set, $s>0$ a non-integer, $T>0$ and $q\in L^p(\Omega )$ with *p* satisfying the restrictions (1.4). Consider the *Runge set*

$$\begin{aligned} \mathscr {R}_W{:}=\left\{ u_\varphi -\varphi \,;\, \varphi \in C^\infty _c (W_T) \right\} \subset L^2(0,T;\widetilde{H}^s(\Omega )), \end{aligned}$$

where $u_\varphi \in C([0,T];H^s({{\mathbb {R}}}^n))\cap C^1([0,T]; L^2({{\mathbb {R}}}^n))$ is the unique solution to (1.6). Then $\mathscr {R}_W$ is dense in $L^2(0,T;\widetilde{H}^s(\Omega ))$.

We call this result optimal Runge approximation as the approximation space coincides with the natural space of solutions in our problem, similarly to the elliptic case, see (ii) on page 2.

With this Runge approximation and a suitable integral identity, similar to the one demonstrated in [27] or [54], we can recover $L^p$-regular linear perturbations, which are not necessarily nonnegative (cf. (a)).

#### Theorem 1.3

Let $\Omega \subset {{\mathbb {R}}}^n$ be a bounded Lipschitz domain, $W_1,W_2\subset \Omega _e$ be nonempty open sets, $s>0$ a non-integer, $T>0$ and $q_j\in L^p(\Omega )$ with *p* satisfying the restrictions (1.4) for $j=1,2$. Let $\Lambda _{q_j}$ be the DN maps of

$$\begin{aligned} {\left\{ \begin{array}{ll} \partial _t^2u +(-\Delta )^su + q_ju=0 & \text { in }\Omega _T,\\ u_j=\varphi & \text { in }(\Omega _e)_T,\\ u_j(0)=\partial _t u_j(0)=0 & \text { in }\Omega , \end{array}\right. } \end{aligned}$$

for $$j=1,2$$, satisfying

1.7 $$\begin{aligned} \Lambda _{q_1}\varphi \big |_{(W_2)_T}=\Lambda _{q_2}\varphi \big |_{(W_2)_T}, \text { for any }\varphi \in C^\infty _c((W_1)_T). \end{aligned}$$

Then there holds $q_1=q_2$ in $\Omega$.

Next, we present our main results on inverse problems for nonlinear nonlocal wave equations. To this purpose, let us introduce two different types of nonlinearities having a polyhomogeneous structure.

#### Definition 1.4

*(Polyhomogeneous nonlinearities)* Let $\Omega \subset {{\mathbb {R}}}^n$ be a bounded domain. Suppose we are given a sequence $\mathfrak {r}{:}=\left( r_k\right) _{k=1}^\infty \subset {{\mathbb {R}}}$ satisfying $0<r_k<r_{k+1}$ for all $k\in {{\mathbb {N}}}$ and let $f:\Omega \times {{\mathbb {R}}}\rightarrow {{\mathbb {R}}}$ be a Carathéodory function.

(i) We call *f* *serially* $\mathfrak {r}$-*polyhomogeneous*, if
  1.8 $$\begin{aligned} f(x,\tau ) = \sum _{k\ge 1} f_k(x,\tau ), \end{aligned}$$
  where each expansion coefficient $f_k:\Omega \times {{\mathbb {R}}}\rightarrow {{\mathbb {R}}}$ is a Carathéodory function satisfying
  1.9 $$\begin{aligned} \left| f_k(x,\tau )\right| \le b_k|\tau |^{r_k+1} \end{aligned}$$
  for some constants $b_k\ge 0$ and is $(r_k+1)$-homogeneous in the $\tau$-variable.
(ii) We call *f* *asymptotically* $\mathfrak {r}$-*polyhomogeneous*, denoted as
  $$ f(x,\tau ) \sim \sum _{k\ge 1} f_k(x,\tau ), $$
  if each expansion coefficient $f_k:\Omega \times {{\mathbb {R}}}\rightarrow {{\mathbb {R}}}$ is a Carathéodory function satisfying (1.9), is $(r_k+1)$-homogeneous in the $\tau$-variable and for all $N\in {{\mathbb {N}}}_{\ge 2}$ there is a constant $C_N>0$ such that
  1.10 $$\begin{aligned} \bigg | f(x,\tau ) - \sum _{k=1}^{N-1} f_k(x,\tau )\bigg | \le C_N |\tau |^{r_N+1} \end{aligned}$$
  for all $|\tau |\le 1$ and $|f(x,\tau )|\le C_\infty |\tau |^{r_\infty +1}$ for all $|\tau |>1$ for a.e. $x\in \Omega$.

With Assumption 1 and Definition 1.4 at hand, we can state our main results for inverse problems of nonlinear nonlocal wave equations.

#### Theorem 1.5

(Recovery of expansion coefficients) Let $\Omega \subset {{\mathbb {R}}}^n$ be a bounded Lipschitz domain, $T>0$, and $s>0$ a non-integer. Let $W_1,W_2\subset \Omega _e$ be nonempty open sets. Suppose the nonlinearities $f^{(j)}$ satisfy the conditions in Assumption 1 with $F^{(1)},F^{(2)}\ge 0$, $a^{(1)},a^{(2)}\in L^{\infty }(\Omega )$, $r^{(1)}=r^{(2)}=r_{\infty }$, and are serially or asymptotically $\mathfrak {r}$-polyhomogeneous such that the corresponding (strictly) monotonically increasing sequence $\mathfrak {r}{:}=(r_k)_{k\in {{\mathbb {N}}}}$ fulfills $\mathfrak {r}\subset (0,r_\infty ]$ (see Definition 1.4). Assume that the DN maps $\Lambda _{f^{(j)}}$ of

$$\begin{aligned} {\left\{ \begin{array}{ll} \partial _t^2u +(-\Delta )^su + f^{(j)}(x,u)=0 & \text { in }\Omega _T,\\ u=\varphi & \text { in }(\Omega _e)_T,\\ u(0)=\partial _t u(0)=0 & \text { in }\Omega , \end{array}\right. } \end{aligned}$$

for $$j=1,2$$, coincide;

1.11 $$\begin{aligned} \Lambda _{f^{(1)}}\varphi \big |_{(W_2)_T}=\Lambda _{f^{(2)}}\varphi \big |_{(W_2)_T} \text { for any }\varphi \in C^\infty _c((W_1)_T). \end{aligned}$$

(i) If $f^{(j)}$ are serially $\mathfrak {r}$-polyhomogeneous with
  1.12 $$\begin{aligned} L_j{:}=\limsup _{k\rightarrow \infty }\frac{b_{k+1}^{(j)}}{b_k^{(j)}}<1, \end{aligned}$$
  then $$f^{(1)}=f^{(2)}$$.
(ii) If $f^{(j)}$ are asymptotically $\mathfrak {r}$-polyhomogeneous such that (1.10) holds for all $\tau \in {{\mathbb {R}}}$ in the cases $2s\le n$, then $f_k^{(1)}(x,\tau )=f_k^{(2)}(x,\tau )$ for all $x\in \Omega$, $\tau \in {{\mathbb {R}}}$ and all $k\in {{\mathbb {N}}}$.

#### Remark 1.6

The earlier article [43] by the authors required nonnegativity of the potential $q\in L^p(\Omega )$ or the stronger assumption that $q\in L^\infty (\Omega )$. This was mainly used for the construction of solutions $u_\varepsilon$, $\varepsilon >0$, to a related parabolically regularized problem, cf. [43, p.15, Proof of Claim 4.2], and for deducing their convergence properties as $\varepsilon \rightarrow 0$. On the inverse problem for the nonlinear equation, the present manuscript generalizes results of [43] from homogeneous to polyhomogeneous nonlinearities, allowing the recovery of the expansion coefficients term-by-term.

### Comparison to inverse problems for local wave equations

The inverse problem of recovering coefficient functions in linear wave equations is classical, and the first results in this direction were by Belishev and Kurylev using a boundary control method [5, 6], see also [22]. Nowadays, there are also results in reconstructing Riemannian manifolds for linear wave and other equations from partial data boundary measurements, such as in [3, 19, 20, 23, 24, 29, 40]. However, in the linear case, these results are based on the boundary control method, which requires the (lower order) coefficient functions to be time-independent or real-analytic in time, see [13]. The recent results in [1, 2] have relaxed the assumptions on the smoothness of the coefficients in the linear local problem under certain curvature bounds, and in particular for metrics close to the Minkowski metric. Thanks to the unique continuation and Runge approximation properties in the nonlocal case, such restrictions are not needed, and one can consider much lower regularity coefficients, such as in Theorem 1.5 (which also includes the linear case, see also [43]). Moreover, nonlocal wave equations often enjoy infinite speed of propagation of the solution, and hence one can recover coefficients in larger domains than in the local case, where causality forces restrictions on the possible domains of reconstruction. There are few results on low-regularity nonlinearities in the local setting, such as [21], where the local elliptic version $-\Delta u + a(x,u)=0$ is considered, and it is proved that a nonlinearity $a(x,\tau )$ which is $L^r(\Omega )$, $r>n/2$, in *x* and $C^{1,\alpha }({{\mathbb {R}}})$ in $\tau$ can be uniquely determined.

Recently, inverse problems for nonlinear (local) wave equations have become mainstream. Let us elaborate on several works in this research field. The nonlinear (self-)interaction of waves will generate new waves, and this effect can be treated as a benefit in solving related inverse problems in the hyperbolic and elliptic settings. The seminal work [26] demonstrated that local measurements can be utilized to recover global topology and differentiable structure uniquely for a semilinear wave equation with a quadratic nonlinearity. Furthermore, general semilinear wave equations on Lorentzian manifolds and related inverse problems were studied for the Einstein-Maxwell equation in [45] and [44], respectively. We also refer readers to [12, 25, 34–37, 53] for different studies, including simultaneous recovery, geometrical inverse problems, and references therein.

### Organization of the paper

The remainder of this paper is arranged as follows. In Section 2, we introduce several function spaces used in this paper. In Section 3, we show that there exists a unique very weak solution to linear nonlocal wave equations. In Section 4, we prove a stronger version of Runge approximation and use it to solve an inverse problem for linear nonlocal wave equations. Finally, we prove Theorem 1.5 in Section 5.

## Preliminaries

In this section, we introduce some notation, which will be used throughout the whole article, and use this occasion to recall several basic facts on fractional Sobolev spaces as well as the fractional Laplacian.

Throughout this article, we use the following convention for the Fourier transform

$$\begin{aligned} \mathcal {F}u(\xi ) {:}=\hat{u}(\xi ){:}= \int _{{{\mathbb {R}}}^n} u(x)e^{-\textrm{i}x \cdot \xi } \,dx \end{aligned}$$

for functions $u:{{\mathbb {R}}}^n\rightarrow {{\mathbb {R}}}$ for example in the Schwartz space $\mathscr {S}({{\mathbb {R}}}^n)$, where $\textrm{i}$ is the imaginary unit. By duality, the Fourier transform can be extended to the space of tempered distributions $\mathscr {S}^{\prime }({{\mathbb {R}}}^n) =(\mathscr {S}({{\mathbb {R}}}^n))^*$, and we use the same notation for it. We denote the inverse Fourier transform by $\mathcal {F}^{-1}$.

For any $s\in {{\mathbb {R}}}$, we let $H^{s}({{\mathbb {R}}}^{n})$ stand for the *fractional Sobolev space*, which consists of all tempered distributions *u* such that

$$\begin{aligned} \Vert u\Vert _{H^{s}(\mathbb {R}^{n})}{:}= \left\| \langle D\rangle ^s u \right\| _{L^2({{\mathbb {R}}}^n)}<\infty , \end{aligned}$$

where $\langle D\rangle ^s$ denotes the Bessel potential of order *s* with the Fourier symbol $\langle \xi \rangle ^s{:}= \left( 1+|\xi |^2\right) ^{s/2}$. We shall also need the following local versions of the fractional Sobolev spaces $H^s({{\mathbb {R}}}^n)$:

$$\begin{aligned} H^{s}(\Omega )&{:}=\left\{ u|_{\Omega } \ ; \, u\in H^{s}(\mathbb {R}^{n})\right\} ,\\ \widetilde{H}^{s}(\Omega )&{:}=\text {closure of } C_{c}^{\infty }(\Omega ) \text { in } H^{s}(\mathbb {R}^{n}), \end{aligned}$$

where $\Omega \subset {{\mathbb {R}}}^n$ is an open set and $H^{s}(\Omega )$ naturally endowed with the quotient norm

$$ \Vert u\Vert _{H^{s}(\Omega )}{:}=\inf \left\{ \Vert U\Vert _{H^{s}(\mathbb {R}^{n})} \ ; \, U\in H^{s}(\mathbb {R}^{n}) \text{ and } U|_{\Omega }=u\right\} . $$

Furthermore, we set

$$ \widetilde{L}^2(\Omega ){:}= \widetilde{H}^0(\Omega )\quad \text {and}\quad \Vert \cdot \Vert _{\widetilde{L}^2(\Omega )}{:}=\Vert \cdot \Vert _{\widetilde{H}^0(\Omega )}=\Vert \cdot \Vert _{L^2({{\mathbb {R}}}^n)}. $$

Let us also emphasize that if $\Omega$ is a Lipschitz domain, then one has the following identification

$$\begin{aligned} (\widetilde{H}^s(\Omega ))^*=H^{-s}(\Omega ). \end{aligned}$$

The *fractional Laplacian* of order $s> 0$ is the homogeneous counterpart of the Bessel potential and hence is the Fourier multiplier

$$\begin{aligned} (-\Delta )^{s} u = \mathcal {F}^{-1}\big ( \left|\xi \right|^{2s}\widehat{u}(\xi )\big ), \text { for }u\in \mathscr {S}({{\mathbb {R}}}^n). \end{aligned}$$

It is not difficult to see that an equivalent norm on $H^s({{\mathbb {R}}}^n)$ is given by

2.1 $$\begin{aligned} \Vert u\Vert _{H^s({{\mathbb {R}}}^n)}^*= \Vert u\Vert _{L^{2}(\mathbb {R}^{n})}+\big \Vert (-\Delta )^{s/2}u \big \Vert _{L^{2}(\mathbb {R}^{n})}, \end{aligned}$$

and the fractional Laplacian is a bounded linear operator as a map $(-\Delta )^{s}:H^{t}({{\mathbb {R}}}^n) \rightarrow H^{t-2s}({{\mathbb {R}}}^n)$ for all $s \ge 0$ and $t \in {{\mathbb {R}}}$. In fact, one can also write $(-\Delta )^s = (-\Delta )^k (-\Delta )^{\alpha }$, where $s=k+\alpha$ with $k=\lfloor s \rfloor \in {{\mathbb {N}}}\cup \{0\}$ and $\alpha =s-k\in (0,1)$.

Using the Hardy–Littlewood–Sobolev lemma and Hölder’s inequality, one can easily see that the following Poincaré inequality holds:

### Proposition 2.1

*(Poincaré inequality (cf. * [50, Lemma 5.4])) Let $\Omega \subset {{\mathbb {R}}}^n$ be a bounded domain. For any $s> 0$, there exists $C>0$ such that

$$\begin{aligned} \Vert u\Vert _{L^2(\Omega )}\le C \big \Vert (-\Delta )^{s/2}u \big \Vert _{L^2({{\mathbb {R}}}^n)}, \text { for any }u\in \widetilde{H}^s(\Omega ). \end{aligned}$$

Taking into account that (2.1) is an equivalent norm on $\widetilde{H}^s(\Omega )$, the Poincaré inequality (Propositions 2.1) ensures the following simple lemma, which will be used throughout the whole article.

### Lemma 2.2

Let $\Omega \subset {{\mathbb {R}}}^n$ be a bounded domain and $s\ge 0$. Then an equivalent norm on $\widetilde{H}^s(\Omega )$ is given by

$$\begin{aligned} \Vert u\Vert _{\widetilde{H}^s(\Omega )}=\big \Vert (-\Delta )^{s/2}u \big \Vert _{L^2({{\mathbb {R}}}^n)}, \end{aligned}$$

which is induced by the inner product

$$\begin{aligned} \langle u,v\rangle _{\widetilde{H}^s(\Omega )}=\big \langle (-\Delta )^{s/2}u,(-\Delta )^{s/2}v \big \rangle _{L^2({{\mathbb {R}}}^n)}. \end{aligned}$$

Finally, let us mention that if *X* is a Banach space, then we denote by $C^k([a,b];X)$ and $L^p(a,b;X)$ ($k\in {{\mathbb {N}}},1\le p\le \infty$) the space of *k*-times continuously differentiable functions and the space of measurable functions $u:(a,b)\rightarrow X$ such that $t\mapsto \Vert u(t)\Vert _X\in L^p([a,b])$, respectively. These Banach spaces are endowed with the norms

$$\begin{aligned} \begin{aligned} \Vert u\Vert _{L^p(a,b\,;X)}&{:}= \bigg (\int _a^b\Vert u(t)\Vert _{X}^p\,dt\bigg )^{1/p}<\infty ,\\ \Vert u\Vert _{C^k([a,b];X)}&{:}= \sup _{0\le \ell \le k}\left\| \partial _t^{\ell }u\right\| _{L^{\infty }(a,b;X)} \end{aligned} \end{aligned}$$

(with the usual modification for $p=\infty$).

## Existence and uniqueness of very weak solutions to linear nonlocal wave equations

The purpose of this section is to extend the well-established theory of *very weak solutions* to linear wave equations in our nonlocal setting. In Section 3.1, we motivate and present the rigorous definition of these solutions. Afterward, in Section 3.2 we formulate a spectral theoretic lemma, which we need later in Section 3.3 for the construction of very weak solutions to NWEQs without a potential term. In Section 3.4, we then establish via a fixed point argument the well-posedness theory of very weak solutions to linear NWEQs with a nonzero potential. Finally, in Section 3.5 we discuss some properties of very weak solutions. In particular, we show that all weak solutions are very weak solutions, which in turn are distributional solutions.

Throughout the whole section, $\Omega \subset {{\mathbb {R}}}^n$ denotes a fixed bounded Lipschitz domain and $s\in {{\mathbb {R}}}_+\setminus {{\mathbb {N}}}$. As usual $\langle \cdot ,\cdot \rangle$ denotes the duality pairing between $\widetilde{H}^s(\Omega )$ and $H^{-s}(\Omega )$. These Hilbert spaces are endowed with the norms $\Vert \cdot \Vert _{\widetilde{H}^s(\Omega )}$, introduced in Lemma 2.2, and

$$\begin{aligned} \Vert G\Vert _{H^{-s}(\Omega )} = \sup \big \{ |\langle G,v\rangle | ; \, v\in \widetilde{H}^s(\Omega ), \, \Vert v\Vert _{\widetilde{H}^s(\Omega )}=1\big \}. \end{aligned}$$

### Definition of very weak solutions

Next, we introduce the notion of very weak solutions to linear NWEQs with possibly a nonzero potential *q*.

#### Definition 3.1

Let $F\in L^2(0,T;H^{-s}(\Omega ))$, $u_0\in \widetilde{L}^2(\Omega )$, $u_1\in H^{-s}(\Omega )$ and $q\in L^{p}(\Omega )$, where the exponent *p* satisfies the restriction (1.4). A function $u:{{\mathbb {R}}}^n_T\rightarrow {{\mathbb {R}}}$ is called a *very weak solution* of

3.1 $$\begin{aligned} {\left\{ \begin{array}{ll} \partial _t^2u +(-\Delta )^su + qu=F & \text { in }\Omega _T,\\ u=0 & \text { in }(\Omega _e)_T,\\ u(0)=u_0, \quad \partial _t u(0)=u_1 & \text { in }\Omega , \end{array}\right. } \end{aligned}$$

if $$u\in C([0,T];\widetilde{L}^2(\Omega ))\cap C^1([0,T];H^{-s}(\Omega ))$$ satisfies

3.2 $$\begin{aligned} \begin{aligned} \int _0^T \left\langle u(t),G(t)\right\rangle _{L^2(\Omega )}\,dt =\int _0^T\left\langle F(t),v(t)\right\rangle _{L^2(\Omega )}\, dt +\left\langle u_1,v(0)\right\rangle -\left\langle u_0,\partial _t v(0)\right\rangle _{L^2(\Omega )}, \end{aligned} \end{aligned}$$

for all $G\in L^2(0,T;\widetilde{L}^2(\Omega ))$, where $v\in C([0,T];\widetilde{H}^s(\Omega ))\cap C^1([0,T];\widetilde{L}^2(\Omega ))$ is the unique (weak) solution of the backward equation

3.3 $$\begin{aligned} {\left\{ \begin{array}{ll} \partial _t^2v +(-\Delta )^sv + qv=G & \text { in }\Omega _T,\\ v=0 & \text { in }(\Omega _e)_T,\\ v(T)=\partial _t v(T)=0 & \text { in }\Omega \end{array}\right. } \end{aligned}$$

(see [43, Theorem 3.1]).

#### Remark 3.2

We recall that if $F\in L^2(0,T;\widetilde{L}^2(\Omega ))$, $u_0\in \widetilde{H}^s(\Omega )$ and $u_1\in \widetilde{L}^2(\Omega )$, then a *weak solution* of (3.1) is a function $u\in C([0,T];\widetilde{H}^s(\Omega ))\cap C^1([0,T];\widetilde{L}^2(\Omega ))$ such that

$$\begin{aligned} \frac{d}{dt}\left\langle \partial _t u,w \right\rangle _{L^2(\Omega )}+ \big \langle (-\Delta )^{s/2}u,(-\Delta )^{s/2}w \big \rangle _{L^2({{\mathbb {R}}}^n)}+\left\langle qu,w \right\rangle _{L^2(\Omega )}=\langle F,w\rangle _{L^2(\Omega )}, \end{aligned}$$

for all $w\in \widetilde{H}^s(\Omega )$ in the sense of $\mathscr {D}^{\prime }((0,T))$. Often, we refer to weak solutions or very weak solutions simply as solutions, because the source term in the relevant PDEs determines which notion of solutions we invoke. Moreover, weak solutions are always very weak solutions, as we will see later in Proposition 3.9.

#### Remark 3.3

Let us emphasize that the restriction on the exponent *p* comes from the observation that if $q\in L^p(\Omega )$ and $u\in C([0,T];L^2(\Omega ))$, then we have $qu\in L^2(0,T;H^{-s}(\Omega ))$ (see (3.33) in the proof of Theorem 3.7).

Before proceeding, let us give a formal motivation for imposing the identity (3.2). We test the equation $\partial _t^2u +(-\Delta )^su + qu=F$ in $H^{-s}(\Omega )$ by the solution $v\in C([0,T];\widetilde{H}^s(\Omega ))$ to (3.3) and integrate the resulting identity from $t=0$ to $t=T$. This gives

3.4 $$\begin{aligned} \begin{aligned}&\, \int _0^T \left\langle \partial _t^2 u(t),v(t)\right\rangle \,dt\\&\quad =-\int _0^T \left\langle (-\Delta )^s u(t),v(t)\right\rangle \,dt -\int _0^T\left\langle qu(t),v(t)\right\rangle \,dt +\int _0^T \langle F(t),v(t)\rangle \,dt\\&\quad =-\int _0^T\left\langle (-\Delta )^s v(t),u(t)\right\rangle \,dt -\int _0^T\left\langle qv(t),u(t)\right\rangle \,dt +\int _0^T \langle F(t),v(t)\rangle \,dt\\&\quad = \int _0^T \left\langle \partial _t^2 v(t),u(t)\right\rangle \,dt - \int _0^T \langle G(t),u(t)\rangle \,dt +\int _0^T \langle F(t),v(t)\rangle \,dt. \end{aligned} \end{aligned}$$

Using an integration by parts, the term on the left-hand side and the first term on the right-hand side can be rewritten as

$$ \begin{aligned} \int _0^T \left\langle \partial _t^2 u(t),v(t)\right\rangle dt&=-\int _0^T \left\langle \partial _t u(t),\partial _t v(t)\right\rangle dt +\left\langle \partial _t u(T),v(T)\right\rangle -\left\langle \partial _t u(0),v(0)\right\rangle \\&=-\int _0^T \left\langle \partial _t u(t),\partial _t v(t)\right\rangle \,dt-\left\langle u_1,v(0)\right\rangle ,\\ \int _0^T \left\langle \partial _t^2 v(t),u(t)\right\rangle dt&=-\int _0^T \left\langle \partial _t v(t),\partial _t u(t)\right\rangle dt +\left\langle \partial _t v(T),u(T)\right\rangle -\left\langle \partial _t v(0),u(0)\right\rangle \\&=-\int _0^T \left\langle \partial _t u(t),\partial _t v(t)\right\rangle \,dt-\left\langle \partial _t v(0),u_0\right\rangle . \end{aligned} $$

Inserting these identities into (3.4), we get (3.2).

#### Remark 3.4

Note that the above computations are only formal because the integration by parts identities require better regularity than $\partial _t u\in L^2(0,T;H^{-s}(\Omega ))$ for the integral $\int _0^T \left\langle \partial _t u(t),\partial _t v(t)\right\rangle \,dt$ to make sense.

### A spectral theoretic lemma

For the construction of very weak solutions to linear nonlocal wave equations, we will need the following elementary spectral theoretic result. Even though the argument is standard, we offer the proof in Appendix A for readers’ convenience.

#### Lemma 3.5

Let $\Omega \subset {{\mathbb {R}}}^n$ be a bounded Lipschitz domain and $s\in {{\mathbb {R}}}_+ \setminus {{\mathbb {N}}}$. There exists a sequence of (Dirichlet) eigenvalues of the fractional Laplacian $(-\Delta )^s$ satisfying $0<\lambda _1\le \lambda _2\le \ldots$ with $\lambda _k\rightarrow \infty$ as $k\rightarrow \infty$ such that the corresponding eigenfunctions $\left( \phi _k\right) _{k\in {{\mathbb {N}}}}\subset \widetilde{H}^s(\Omega )$ have the following properties:

(i) $\left( \phi _k\right) _{k\in {{\mathbb {N}}}}$ is an orthonormal basis of $\widetilde{L}^2(\Omega )$,
(ii) $\big (\lambda _k^{-1/2}\phi _k\big )_{k\in {{\mathbb {N}}}}$ is an orthonormal basis of $\widetilde{H}^s(\Omega )$,
(iii) $\big ( \lambda _k^{1/2}\phi _k\big )_{k\in {{\mathbb {N}}}}$ is an orthonormal basis of $H^{-s}(\Omega )$.

### Very weak solutions to linear nonlocal wave equations without potential

The main purpose of this section is to prove the following well-posedness result.

#### Theorem 3.6

(Well-posedness of NWEQ with $q=0$) Let $F\in L^2(0,T;H^{-s}(\Omega ))$, $u_0\in \widetilde{L}^2(\Omega )$ and $u_1\in H^{-s}(\Omega )$. Then there exists a unique solution of

3.5 $$\begin{aligned} {\left\{ \begin{array}{ll} \partial _t^2u +(-\Delta )^su =F & \text { in }\Omega _T,\\ u=0 & \text { in }(\Omega _e)_T,\\ u(0)=u_0, \quad \partial _t u(0)=u_1 & \text { in }\Omega . \end{array}\right. } \end{aligned}$$

Moreover, the following continuity estimate holds

3.6 $$\begin{aligned} \begin{aligned} \Vert u(t)\Vert _{L^2(\Omega )}+\left\| \partial _t u(t)\right\| _{H^{-s}(\Omega )}\le C\big (\left\| u_0\right\| _{L^2(\Omega )}+\left\| u_1\right\| _{H^{-s}(\Omega )}+\Vert F\Vert _{L^2(0,T;H^{-s}(\Omega ))}\big ), \end{aligned} \end{aligned}$$

for some $C>0$ and for all $0\le t\le T$.

#### Proof

We use the Fourier method to show the existence of a solution to (3.5), that is we make the ansatz

3.7 $$\begin{aligned} u(t)=\sum _{k=1}^{\infty }c_k(t)\phi _k \end{aligned}$$

and for later convenience we set

$$ u_m(t){:}=\sum _{k=1}^m c_k(t)\phi _k, $$

for any $m\in {{\mathbb {N}}}$. For *u* to satisfy (3.5) in $H^{-s}(\Omega )$, the coefficient $c_k$, $k\in {{\mathbb {N}}}$, needs to solve the initial value problem

3.8 $$\begin{aligned} {\left\{ \begin{array}{ll} c''_k(t)+\lambda _k c_k(t)=F_k(t), \\ c_k(0)=u_0^k, \, c'_k(0)=u_1^k \end{array}\right. } \end{aligned}$$

for $0<t<T$, where we set

$$ u_0^k=\left\langle u_0,\phi _k\right\rangle _{L^2(\Omega )},\, u_1^k=\left\langle u_1,\phi _k\right\rangle \text { and }F_k(t)=\left\langle F(t),\phi _k\right\rangle . $$

By Duhamel’s principle, for any $k\in {{\mathbb {N}}}$, the coefficients $c_k$ are given by

3.9 $$\begin{aligned} \begin{aligned} c_k(t)&=u_0^k\cos \big (\lambda _k^{1/2}t\big )+\lambda _k^{-1/2}u_1^k \sin \big (\lambda _k^{1/2}t\big ) \\&\qquad +\lambda _k^{-1/2}\int _0^tF_k(\tau )\sin \big (\lambda _k^{1/2}(t-\tau )\big )\,d\tau . \end{aligned} \end{aligned}$$

*Step 1.* We first show that for any $t\in [0,T]$, the series in (3.7) converges in $\widetilde{L}^2(\Omega )$. By [7, Corollary 5.10], we only need to ensure that $c_k(t)\in \ell ^2$. By Jensen’s inequality, we may estimate

3.10 $$\begin{aligned} \begin{aligned} |c_k(t)|^2&\le 3\bigg (|u_0^k|^2+\lambda _k^{-1}|u_1^k|^2+\lambda _k^{-1}\left| \int _0^t F_k(\tau )\sin \big (\lambda _k^{1/2}(t-\tau )\big )\,d\tau \right| ^2\bigg )\\&\le 3\bigg (|u_0^k|^2+\lambda _k^{-1}|u_1^k|^2+t\lambda _k^{-1}\int _0^t |F_k(\tau )|^2\,d\tau \bigg ), \end{aligned} \end{aligned}$$

for any $k\in {{\mathbb {N}}}$. As $u_0\in \widetilde{L}^2(\Omega )$ and $(\phi _k)_{k\in {{\mathbb {N}}}}$ is an orthonormal basis in $\widetilde{L}^2(\Omega )$, [7, Corollary 5.10] implies $(u_0^k)_{k\in {{\mathbb {N}}}}\in \ell ^2$ with

3.11 $$\begin{aligned} \Vert u_0\Vert _{L^2(\Omega )}^2=\sum _{k=1}^{\infty }|u_0^k|^2. \end{aligned}$$

Similarly, we know by (A.12) that $(\lambda _k^{-1/2}u_1^k)_{k\in {{\mathbb {N}}}}\in \ell ^2$ with

3.12 $$\begin{aligned} \Vert u_1\Vert _{H^{-s}(\Omega )}^2=\sum _{k=1}^{\infty }\lambda _k^{-1}|u_1^k|^2. \end{aligned}$$

On the other hand, the formula (A.12) shows that if $G\in L^2(0,T;H^{-s}(\Omega ))$, then $(\lambda _k^{-1}G_k)_{k\in {{\mathbb {N}}}}\in L^2(0,T;\ell ^2)$. Additionally, by Tonelli’s theorem, the integral and sum can be exchanged so that

3.13 $$\begin{aligned} \Vert G\Vert _{L^2(0,T;H^{-s}(\Omega ))}^2=\Vert \lambda _k^{-1/2}G_k\Vert _{L^2(0,T;\ell ^2)}^2=\sum _{k=1}^{\infty }\lambda _k^{-1}\int _0^T |G_k(t)|^2\,dt. \end{aligned}$$

This estimate can be applied to $F\in L^2(0,T;H^{-s}(\Omega ))$. Hence, to sum up, we have

$$\begin{aligned} \Vert c_k(t)\Vert ^2_{\ell ^2}\le 3\big ( \Vert u_0\Vert _{L^2(\Omega )}^2+\Vert u_1\Vert _{H^{-s}(\Omega )}^2+T\Vert F\Vert _{L^2(0,T;H^{-s}(\Omega ))}^2\big ). \end{aligned}$$

Now, again invoking [7, Corollary 5.10] and Lemma 3.5, we deduce (3.7) converges in $\widetilde{L}^2(\Omega )$ for any $t\in [0,T]$ and

3.14 $$\begin{aligned} \Vert u(t)\Vert _{L^2(\Omega )}^2=\Vert c_k(t)\Vert _{\ell ^2}^2\le 3\big (\Vert u_0\Vert _{L^2(\Omega )}^2+\Vert u_1\Vert _{H^{-s}(\Omega )}^2+T\Vert F\Vert _{L^2(0,T;H^{-s}(\Omega ))}^2\big ). \end{aligned}$$

*Step 2.* We first show that $u\in C([0,T];\widetilde{L}^2(\Omega ))$. To see this, it is enough to show that $u_m\in C([0,T];\widetilde{L}^2(\Omega ))$ for $m\in {{\mathbb {N}}}$ and $u_m\rightarrow u$ in $\widetilde{L}^2(\Omega )$ as $m\rightarrow \infty$ uniformly in $0\le t\le T$. Note that we have

$$ \left\| u_m(t)-u_m(t')\right\| _{L^2(\Omega )}\le \sum _{k=1}^m \left| c_k(t)-c_k(t')\right| , $$

for $t,t'\in [0,T]$ and so $u_m\in C([0,T];\widetilde{L}^2(\Omega ))$ as long as $c_k\in C([0,T])$. The first two terms of $c_k$ (see (3.9)) are continuous, hence it only remains to show that

3.15 $$\begin{aligned} d_k(t){:}=\lambda _k^{-1/2}\int _0^t F_k(\tau )\sin \big (\lambda _k^{1/2}(t-\tau )\big )\,d\tau \in C([0,T]). \end{aligned}$$

Let us suppose that $t\ge t'$. Then we may calculate

3.16 $$\begin{aligned} \begin{aligned}&\, \left| d_k(t)-d_k(t')\right| \\&\quad =\lambda _k^{-1/2}\left| \int _0^t F_k(\tau )\sin \big (\lambda _k^{1/2}(t-\tau )\big )\,d\tau -\int _0^{t'} F_k(\tau )\sin \big (\lambda _k^{1/2}(t'-\tau )\big )\,d\tau \right| \\&\quad \le \lambda _k^{-1/2}\int _{t'}^t |F_k(\tau )|\big |\sin \big (\lambda _k^{1/2}(t-\tau )\big )\big |\,d\tau \\&\qquad \, +\lambda _k^{-1/2}\int _0^{t'}|F_k(\tau )|\big |\sin \big (\lambda _k^{1/2}(t'-\tau )\big )-\sin \big (\lambda _k^{1/2}(t-\tau )\big )\big |\,d\tau \\&\quad \le \lambda _k^{-1/2}\int _{t'}^t |F_k(\tau )|\,d\tau \\&\qquad \, +\lambda _k^{-1/2}\int _0^{t'}|F_k(\tau )|\big |\sin \big (\lambda _k^{1/2}(t'-\tau )\big )-\sin \big (\lambda _k^{1/2}(t-\tau )\big )\big |\,d\tau \\&\quad \le |t-t'|^{1/2}\bigg (\int _{t'}^t \lambda _k^{-1}|F_k(\tau )|^2\,d\tau \bigg )^{1/2}\\&\qquad \,+\bigg (\int _0^{t'}\lambda _k^{-1}|F_k(\tau )|^2\,d\tau \bigg )^{1/2}\bigg (\int _0^{t'}\big |\sin \big (\lambda _k^{1/2}(t'-\tau )\big )-\sin \big (\lambda _k^{1/2}(t-\tau )\big )\big |^2\,d\tau \bigg )^{1/2}\\&\quad \le |t-t'|^{1/2}\Vert F\Vert _{L^2(0,T;H^{-s}(\Omega ))}\\&\qquad \, +\Vert F\Vert _{L^2(0,T;H^{-s}(\Omega ))}\bigg (\int _0^{t'}|\sin (\lambda _k^{1/2}(t'-\tau ))-\sin (\lambda _k^{1/2}(t-\tau ))|^2\,d\tau \bigg )^{1/2}. \end{aligned} \end{aligned}$$

In the first inequality, we wrote

$$ \sin \big (\lambda _k^{1/2}(t'-\tau )\big )=\big (\sin \big (\lambda _k^{1/2}(t'-\tau )\big )-\sin \big (\lambda _k^{1/2}(t-\tau )\big )\big )+\sin \big (\lambda _k^{1/2}(t'-\tau )\big ) $$

and in the fourth inequality used (3.13). Now, let $\epsilon >0$ and choose first $\rho >0$ such that $\rho ^{1/2}\Vert F\Vert _{L^2(0,T;H^{-s}(\Omega ))}<\epsilon /2$. Then choose $\delta >0$ such that

$$ \Vert F\Vert _{L^2(0,T;H^{-s}(\Omega ))}T^{1/2}\delta <\epsilon /2. $$

By uniform continuity of the sine function, we can find $\eta >0$ such that $|\sin x-\sin y|<\delta$, whenever $|x-y|<\eta$. Now, we set

$$ \mu {:}=\min (\rho ,\eta /\lambda _k^{1/2}). $$

The above choices show that if $|t-t'|<\mu$ and $t\ge t'$, then

$$ \begin{aligned} \left| d_k(t)-d_k(t')\right|&\le \rho ^{1/2}\Vert F\Vert _{L^2(0,T;H^{-s}(\Omega ))}+\Vert F\Vert _{L^2(0,T;H^{-s}(\Omega ))}\delta (t')^{1/2}\\&<\epsilon /2+\Vert F\Vert _{L^2(0,T;H^{-s}(\Omega ))}\delta T^{1/2}\\&<\epsilon . \end{aligned} $$

Interchanging the roles of *t* and $t'$ shows that $d_k$ is uniformly continuous because the constant does not depend on the particular point *t* or $t'$ (but the choice of $\mu$ depends on *k*). Hence, we have shown that $u_m\in C([0,T];\widetilde{L}^2(\Omega ))$.

Next, we prove that $$u_m $$ converges uniformly to *u* in $$\widetilde{L}^2(\Omega )$$ on [0, *T*] as $$m\rightarrow \infty $$. Let $$\ell \ge m$$, then by (3.10) we deduce that

$$ \begin{aligned} \left\| u_\ell (t)-u_m(t)\right\| _{L^2(\Omega )}^2&=\bigg \Vert \sum _{k=m+1}^\ell c_k(t)\phi _k\bigg \Vert _{L^2(\Omega )}^2 \\&=\sum _{k=m+1}^\ell \left| c_k(t)\right| ^2\\&\le C\sum _{k=m+1}^\ell \bigg (\left| u_0^k\right| ^2+\lambda _k^{-1}\left| u_1^k\right| ^2+T\lambda _k^{-1}\int _0^T \left| F_k(\tau )\right| ^2\,d\tau \bigg ), \end{aligned} $$

for some constant $C>0$. Passing to the limit $\ell \rightarrow \infty$ gives

$$ \left\| u(t)-u_m(t)\right\| _{L^2(\Omega )}^2\le C\sum _{k=m+1}^{\infty } \bigg ( \left| u_0^k \right| ^2+\lambda _k^{-1}\left| u_1^k\right| ^2+T\lambda _k^{-1}\int _0^T \left| F_k(\tau )\right| ^2\,d\tau \bigg ) $$

for any $m\in {{\mathbb {N}}}$. The right-hand side is independent of $t\in [0,T]$ and by summability of the right-hand side (see (3.11), (3.12) and (3.13)) it needs to go to zero as *m* tends to infinity. Thus, the convergence $u_m\rightarrow u$ in $\widetilde{L}^2(\Omega )$ as $m\rightarrow \infty$ is uniform in $t\in [0,T]$.

*Step 3.* Let us show that $u\in C^1([0,T];H^{-s}(\Omega ))$. We first establish that $u_m\in C^1([0,T];H^{-s}(\Omega ))$ for any $m\in {{\mathbb {N}}}$. Formally, by differentiating $c_k$, one may compute

3.17 $$\begin{aligned} \begin{aligned} c'_k(t)&=-\lambda _k^{1/2}u_0^k\sin \big (\lambda _k^{1/2}t\big )+u_1^k\cos \big (\lambda _k^{1/2}t\big )+\int _0^t F_k(\tau )\cos \big (\lambda _k^{1/2}(t-\tau )\big )\,d\tau , \end{aligned} \end{aligned}$$

for any $k\in {{\mathbb {N}}}$. Taking derivatives for the first two terms does not cause any difficulty, but for the integral term, we need to justify it. Using the definition of $d_k$ from (3.15), for any $t\in [0,T]$ and $h>0$ such that $t+h\in [0,T]$, one can compute

$$ \begin{aligned}&\quad \, \bigg |\frac{d_k(t+h)-d_k(t)}{h}-\int _0^tF_k(\tau )\cos \big (\lambda _k^{1/2}(t-\tau )\big )\,d\tau \bigg |\\&=\bigg |\int _t^{t+h}\lambda _k^{-1/2}F_k(\tau )\frac{\sin \big (\lambda _k^{1/2}(t+h-\tau )\big )}{h}\,d\tau \\&\quad \,\, +\int _0^t\lambda _k^{-1/2}F_k(\tau ) \\&\qquad \quad \cdot \bigg (\frac{\sin \big (\lambda _k^{1/2}(t+h-\tau )\big )-\sin \big (\lambda _k^{1/2}(t-\tau )\big )}{h}-\lambda _k^{1/2}\cos \big (\lambda _k^{1/2}(t-\tau )\big )\bigg )\,d\tau \bigg |\\&\le \int _t^{t+h}\lambda _k^{-1/2}|F_k(\tau )|\bigg |\frac{\sin \big (\lambda _k^{1/2}(t+h-\tau )\big )}{h}\bigg |\,d\tau \\&\quad \, +\int _0^t\lambda _k^{-1/2}\left| F_k(\tau )\right| \\&\qquad \quad \cdot \bigg |\frac{\sin \big (\lambda _k^{1/2}(t+h-\tau )\big )-\sin \big (\lambda _k^{1/2}(t-\tau )\big )}{h}-\lambda _k^{1/2}\cos \big (\lambda _k^{1/2}(t-\tau )\big )\bigg |\,d\tau \\&={:}I_h+II_h. \end{aligned} $$

Next, we show that both expressions $I_h$ and $II_h$ vanish as $h\rightarrow 0$.

For $I_h$, by the triangle inequality, we have

$$ \begin{aligned} I_h&\le \int _t^{t+h}\lambda _k^{-1/2}\left| F_k(\tau )\right| \bigg |\frac{\sin \big (\lambda _k^{1/2}(t+h-\tau )\big )-\sin \big (\lambda _k^{1/2}(t-\tau )\big )}{h}\bigg |\,d\tau \\&\quad \, +\underbrace{\int _t^{t+h}\lambda _k^{-1/2}\left| F_k(\tau )\right| \bigg |\frac{\sin \big (\lambda _k^{1/2}(t-\tau )\big )}{h}\bigg | d\tau }_{=\frac{1}{h}\int _t^{t+h}\lambda _k^{-1/2}\left| F_k(\tau )\right| |\sin (\lambda _k^{1/2}(t-\tau ))| d\tau }\\&=\int _t^{t+h}\lambda _k^{-1/2}\left| F_k(\tau )\right| \bigg |\frac{\sin \big (\lambda _k^{1/2}(t+h-\tau )\big )-\sin \big (\lambda _k^{1/2}(t-\tau )\big )}{h}\bigg |\,d\tau +o(1)\\&=\int _t^{t+h}\left| F_k(\tau )\right| \big |\cos \big (\lambda _k^{1/2}(\eta -\tau )\big )\big |d\tau +o(1)\\&\le \int _t^{t+h}\left| F_k(\tau )\right| \,d\tau +o(1)\\&=o(1) \end{aligned} $$

as $h\rightarrow 0$. In the first equality we used that $F_k\in L^2((0,T))$ and Lebesgue’s differentiation theorem implies

$$ \frac{1}{h}\int _t^{t+h}\lambda _k^{-1/2}\left| F_k(\tau )\right| \big |\sin (\lambda _k^{1/2}(t-\tau ))\big | d\tau \rightarrow 0 \text { as }h\rightarrow 0. $$

In the second equality we applied the mean value theorem, where $\eta \in (t,t+h)$, and in the last equality the absolute continuity of the Lebesgue integral.

On the other hand, the fact that $II_h\rightarrow 0$ as $h\rightarrow 0$ is a simple application of Lebesgue’s dominated convergence theorem. The same argument works out for $h<0$ and hence the $d_k$ is differentiable with the derivative

3.18 $$\begin{aligned} d'_k(t)=\int _0^tF_k(\tau )\cos \big (\lambda _k^{1/2}(t-\tau )\big )\,d\tau . \end{aligned}$$

Hence, we have proved the formula (3.17).

It remains to show that $u'_m\in C([0,T];H^{-s}(\Omega ))$, but by the same argument as above it is enough to establish $c'_k\in C([0,T])$. The first two terms in $c'_k$ are clearly continuous and hence we only need to show the continuity of $d'_k$ given by the formula (3.18). Let $t\ge t'$. By the same computation as in (3.16) up to replacing $\sin$ by $\cos$ and forgetting the prefactor $\lambda _k^{-1/2}$, we have

$$ \begin{aligned} \left| d'_k(t)-d'_k(t')\right|&\le \int _{t'}^t |F_k(\tau )|\,d\tau \\&\quad \, +\int _0^{t'}\left| F_k(\tau )\right| \big |\cos \big (\lambda _k^{1/2}(t'-\tau )\big )-\cos \big (\lambda _k^{1/2}(t-\tau )\big )\big |d\tau . \end{aligned} $$

Now, let $\epsilon >0$. As $F_k\in L^2((0,T))$, by the absolute continuity of the Lebesgue integral, we can find $\delta >0$ such that the first term is smaller than $\epsilon /2$, whenever $|t-t'|<\delta$. On the other hand, we can find a $\rho >0$ such that there holds

$$ |\cos x-\cos y|<\epsilon /2\Vert F_k\Vert _{L^1((0,T))} $$

whenever $$|x-y|<\rho $$. Let

$$ \mu =\min (\delta ,\rho /\lambda _k^{1/2}). $$

Hence, if $|t-t'|<\mu$, then we have

$$ \big |\lambda _k^{1/2}(t'-\tau )-\lambda _k^{1/2}(t-\tau )\big |=\lambda _k^{1/2}|t-t'|<\rho $$

and hence

$$ \left| d'_k(t)-d'_k(t')\right|<\epsilon /2+ \frac{\epsilon }{2\Vert F_k\Vert _{L^1((0,T))}}\int _0^{t'}|F_k(\tau )|\,d\tau <\epsilon . $$

The very same argument holds when $t\le t'$, and as all parameters $\delta ,\rho ,\mu$ are independent of *t* and $t'$, we have shown that $d'_k$ are uniformly continuous on [0, *T*]. Hence, we have $u_m\in C^1([0,T];H^{-s}(\Omega ))$ for all $m\in {{\mathbb {N}}}$.

Now, by Lemma 3.5 and the same arguments as in (3.10), we get

3.19 $$\begin{aligned} \begin{aligned} \left\| u'_m(t)\right\| ^2_{H^{-s}(\Omega )}&=\bigg \Vert \sum _{k=1}^m c'_k(t)\phi _k\bigg \Vert _{H^{-s}(\Omega )}^2\\&=\bigg \Vert \sum _{k=1}^m \lambda _k^{-1/2}c'_k(t)(\lambda _k^{1/2}\phi _k)\bigg \Vert _{H^{-s}(\Omega )}^2\\&=\sum _{k=1}^m\lambda _k^{-1}|c'_k(t)|^2\\&\le 3\sum _{k=1}^m\lambda _k^{-1}\bigg (\lambda _k|u_0^k|^2+|u_1^k|^2+\bigg (\int _0^t|F_k(\tau )|\,d\tau \bigg )^2\bigg )\\&\le 3\sum _{k=1}^m\bigg (|u_0^k|^2+\lambda _k^{-1}|u_1^k|^2+T\lambda _k^{-1}\int _0^T|F_k(\tau )|^2\,d\tau \bigg ). \end{aligned} \end{aligned}$$

Using (3.11), (3.12) and (3.13), this implies

3.20 $$\begin{aligned} \left\| u'_m(t)\right\| _{H^{-s}(\Omega )}^2\le 3 \big (\Vert u_0\Vert _{L^2(\Omega )}^2+\Vert u_1\Vert _{H^{-s}(\Omega )}^2+T\Vert F\Vert _{L^2(0,T;H^{-s}(\Omega )}^2\big ). \end{aligned}$$

Furthermore, we have

3.21 $$\begin{aligned} \begin{aligned} \left\| u'_\ell (t)-u'_m(t)\right\| ^2_{H^{-s}(\Omega )}&= \sum _{k=m+1}^\ell \lambda _k^{-1}|c'_k(t)|^2\le \sum _{k=m+1}^{\infty }\lambda _k^{-1}|c'_k(t)|^2 \end{aligned} \end{aligned}$$

for all $\ell \ge m$. Observing that the right-hand side goes to zero as $m\rightarrow \infty$, we see that $( u_m(t))_{m\in {{\mathbb {N}}}}\subset H^{-s}(\Omega )$ is a Cauchy sequence in $H^{-s}(\Omega )$ and therefore converges to some unique limit $w(t) \in H^{-s}(\Omega )$. Passing to the limit $\ell \rightarrow \infty$ in (3.21) and using the estimates from (3.19), we get

$$ \left\| w(t)-u'_m(t)\right\| _{H^{-s}(\Omega )}^2\le 3\sum _{k=m+1}^{\infty }\bigg (|u_0^k|^2+\lambda _k^{-1}|u_1^k|^2+T\lambda _k^{-1}\int _0^T|F_k(\tau )|^2\,d\tau \bigg ) $$

for any $m\in {{\mathbb {N}}}$. The sum on the right-hand side is independent of *t* and hence the convergence $u'_m\rightarrow w$ in $H^{-s}(\Omega )$ as $m\rightarrow \infty$ is uniform in $t\in [0,T]$. It is well-known that this implies $u\in C^1([0,T];H^{-s}(\Omega ))$ with $u'=w$. Furthermore, by (3.20) there holds

$$\begin{aligned} \Vert u'\Vert _{L^{\infty }(0,T;H^{-s}(\Omega ))}\le C \left( \Vert u_0\Vert _{L^2(\Omega )}+\Vert u_1\Vert _{H^{-s}(\Omega )}+\Vert F\Vert _{L^2(0,T;H^{-s}(\Omega )}\right) , \end{aligned}$$

for some $C>0$. Notice that this estimate, together with (3.14) establishes (3.6).

*Step 4.* In this step, we show that *u* is in fact a solution of (3.5). First, let us note that by formally applying the Leibniz rule and (3.9), one has

3.22 $$\begin{aligned} \begin{aligned} c''_k(t)&=-\lambda _ku_0^k \cos \big (\lambda _k^{1/2}t\big )-\lambda _k^{1/2}u_1^k\sin \big (\lambda _k^{1/2}t\big )+F_k(t)\\&\quad \,-\lambda _k^{1/2}\int _0^t F_k(\tau )\sin \big (\lambda _k^{1/2}(t-\tau )\big )\,d\tau \\&=-\lambda _k c_k(t)+F_k(t). \end{aligned} \end{aligned}$$

Thus, $c_k$ indeed solves (3.8). To see that the first equality sign in formula (3.22) holds, it is enough to show that $d'_k$ is differentiable with derivative

3.23 $$\begin{aligned} d''_k(t)=F_k(t)-\lambda _k^{1/2}\int _0^t F_k(\tau )\sin \big (\lambda _k^{1/2}(t-\tau )\big )\,d\tau . \end{aligned}$$

We can repeat the same computation as for the first derivative. This time we have

$$ \begin{aligned}&\quad \, \bigg |\frac{d'_k(t+h)-d'_k(t)}{h}-F_k(t)+\lambda _k^{1/2}\int _0^tF_k(\tau )\sin \big (\lambda _k^{1/2}(t-\tau )\big )\,d\tau \bigg |\\&\le \int _t^{t+h}\bigg |\frac{F_k(\tau )\cos \big (\lambda _k^{1/2}(t+h-\tau )\big )-F_k(t)}{h}\bigg |\,d\tau \\&\quad \, +\int _0^t|F_k(\tau )|\bigg |\frac{\cos \big (\lambda _k^{1/2}(t+h-\tau )\big )-\cos \big (\lambda _k^{1/2}(t-\tau )\big )}{h}-\lambda _k^{1/2}\sin \big (\lambda _k^{1/2}(t-\tau )\big )\bigg |\,d\tau \\&= {:}III_h+IV_h. \end{aligned} $$

The second term $IV_h$ again goes to zero by Lebesgue’s dominated convergence theorem. For the first term $III_h$, we proceed similarly to *I* above. This gives

$$ \begin{aligned} III_h&\le \int _t^{t+h}|F_k(\tau )|\bigg |\frac{\cos \big (\lambda _k^{1/2}(t+h-\tau )\big )-\cos \big (\lambda _k^{1/2}(t-\tau )\big )}{h}\bigg |\,d\tau \\&\quad \, +\int _t^{t+h}\bigg |\frac{F_k(\tau )\cos \big (\lambda _k^{1/2}(t-\tau )\big )-F_k(t)}{h}\bigg |\,d\tau . \end{aligned} $$

The second term goes to zero as $h\rightarrow 0$ by Lebesgue’s differentiation theorem, and for the first term, one can use the mean value theorem and the absolute continuity of the integral to find $III_h\rightarrow 0$ as $h\rightarrow 0$. This proves (3.23) and hence (3.22). From the differential equation for $c_k$ we get $c''_k\in L^2((0,T))$. Note that as *F* is not (in general) continuous, we generally do not have $c_k\in C^2([0,T])$. But in fact $c'_k\in H^1((0,T))$ and the fundamental theorem of calculus imply $c_k \in C^{1,1/2}([0,T])$.

Now, we wish to show that *u* is a solution in the sense of Definition 3.1. To this end, let us assume that $G\in L^2(0,T;\widetilde{L}^2(\Omega ))$ and $v\in C([0,T];\widetilde{H}^s(\Omega ))\cap C^1([0,T];\widetilde{L}^2(\Omega ))$ is the unique solution to

3.24 $$\begin{aligned} {\left\{ \begin{array}{ll} \partial _t^2v +(-\Delta )^sv =G & \text { in }\Omega _T,\\ v=0 & \text { in }(\Omega _e)_T,\\ v(T)= \partial _t v(T)=0 & \text { in }\Omega . \end{array}\right. } \end{aligned}$$

Note that from this equation we also have $\partial _t^2 v\in L^2(0,T;H^{-s}(\Omega ))$. By Lemma 3.5 we can write

$$\begin{aligned} v(t)=\sum _{k=1}^{\infty }\alpha _k(t)\phi _k, \end{aligned}$$

where $\alpha _k=\langle v(t),\phi _k\rangle _{L^2(\Omega )}$. Furthermore, note that by our choice of $\phi _k$ and the inner product on $H^{-s}(\Omega )$ (see (A.16)), there holds

$$\begin{aligned} \alpha _k=\lambda _k^{-1/2}\big \langle v(t),\lambda _k^{-1/2}\phi _k\big \rangle _{\widetilde{H}^s(\Omega )}=\lambda _k^{1/2}\big \langle v(t),\lambda _k^{1/2}\phi _k\big \rangle _{H^{-s}(\Omega )}. \end{aligned}$$

The last equality is shown in (A.17). For later convenience, we let $v_m$ be defined via

$$ v_m(t)=\sum _{k=1}^m \alpha _k(t)\phi _k. $$

Moreover, we know that:

- For any $t\in [0,T]$ one has $v_m(t)\rightarrow v(t)$ in $\widetilde{H}^s(\Omega )$ as $m\rightarrow \infty$.
- There holds $\alpha _k\in C^1([0,T])\cap H^2((0,T))$ for any $k\in {{\mathbb {N}}}$.
- For any $k\in {{\mathbb {N}}}$ the functions $\alpha _k$ solve
  $$\begin{aligned} {\left\{ \begin{array}{ll} \alpha ''_k(t)+\lambda _k \alpha _k(t)=G_k(t)\\ \alpha _k(T)=\alpha '_k(T)=0, \end{array}\right. } \end{aligned}$$
  for $$0<t<T$$, where $$G_k(t)=\langle G(t),\phi _k\rangle _{L^2(\Omega )}$$.

In fact, (a) follows from Lemma 3.5 and the regularity of *v*. The regularity $\alpha _k\in C^1([0,T])$ in (b) follows from $v\in C^1([0,T];\widetilde{L}^2(\Omega ))$. The claim that $\alpha _k\in H^2((0,T))$ can be seen as follows. First of all, $v''\in L^2(0,T;H^{-s}(\Omega ))$ implies

$$ -\int _0^T v'(t)\eta '(t)\,dt=\int _0^Tv''(t)\eta (t)\,dt\text { in } H^{-s}(\Omega ) $$

for all $\eta \in C_c^{\infty }((0,T))$. Acting on this identity by $w\mapsto \lambda _k^{1/2}\langle w,\lambda _k^{1/2}\phi _k\rangle _{H^{-s}(\Omega )}\in (H^{-s}(\Omega ))^*$ gives

$$ -\int _0^T\lambda _k^{1/2}\langle v'(t),\lambda _k^{1/2}\phi _k\rangle _{H^{-s}(\Omega )}\eta '(t)\,dt=\int _0^T \lambda _k^{1/2}\langle v''(t),\lambda _k^{1/2}\phi _k\rangle _{H^{-s}(\Omega )}\eta (t)\,dt. $$

Now, the first factor on the left-hand side is nothing else than $\alpha '_k$, and thus

$$ \alpha ''_k(t)=\lambda _k^{1/2}\langle v''(t),\lambda _k^{1/2}\phi _k\rangle _{H^{-s}(\Omega )}\in L^2((0,T)). $$

This shows that $\alpha _k\in H^2((0,T))$ for $k\in {{\mathbb {N}}}$. The endpoint conditions in (c) follow from $v(T)=v'(T)=0$. From the previous calculation, (A.16) and (3.24), we can compute

$$ \begin{aligned} \alpha ''_k(t)&=\lambda _k^{1/2}\big \langle v''(t),\lambda _k^{1/2}\phi _k\big \rangle _{H^{-s}(\Omega )}\\&=\lambda _k^{1/2}\big \langle v''(t)-G(t),\lambda _k^{1/2}\phi _k\big \rangle _{H^{-s}(\Omega )}+\lambda _k^{1/2}\big \langle G(t),\lambda _k^{1/2}\phi _k\big \rangle _{H^{-s}(\Omega )}\\&=\big \langle S(v''(t)-G(t)),S(\lambda _k\phi _k)\big \rangle _{\widetilde{H}^s(\Omega )}+\left\langle S(G(t)),S(\lambda _k\phi _k)\right\rangle _{\widetilde{H}^s(\Omega )}\\&=-\big \langle v(t),\phi _k\big \rangle _{\widetilde{H}^s(\Omega )}+\left\langle S(G(t)),\phi _k\right\rangle _{\widetilde{H}^s(\Omega )}\\&=-\lambda _k\langle v(t),\phi _k\rangle _{L^2(\Omega )}+\langle G(t),\phi _k\rangle _{L^2(\Omega )}\\&=-\lambda _k\alpha _k(t)+G_k(t), \end{aligned} $$

where $S:H^{-s}(\Omega )\rightarrow \widetilde{H}^s(\Omega )$ is the source-to-solution map for the Dirichlet problem of the fractional Laplacian $(-\Delta )^s$ (see Appendix  A for more details). This verifies that $\alpha _k$ satisfies (c) . By $c_k\in C^1([0,T])\cap H^2((0,T))$, (3.8), (b) and (c) we may calculate

3.25 $$\begin{aligned} \begin{aligned} \int _0^T c''_k \alpha _k\,dt&=-\int _0^T c'_k\alpha '_k\,dt+c'_k(T)\alpha _k(T)-c'_k(0)\alpha _k(0)\\&=-\int _0^T c'_k\alpha '_k\,dt-u_1^k\alpha _k(0) \end{aligned} \end{aligned}$$

and

3.26 $$\begin{aligned} \begin{aligned} \int _0^T c_k \alpha ''_k\,dt&=-\int _0^T c'_k\alpha '_k\,dt+c_k(T)\alpha '_k(T)-c_k(0)\alpha '_k(0)\\&=-\int _0^T c'_k\alpha '_k\,dt-u_0^k\alpha '_k(0). \end{aligned} \end{aligned}$$

Inserting (3.26) into (3.25) yields

$$\begin{aligned} \int _0^T c''_k \alpha _k\,dt=\int _0^T c_k\alpha ''_k\,dt+u_0^k\alpha '_k(0)-u_1^k\alpha _k(0). \end{aligned}$$

Hence by (3.8) and (c), we get

3.27 $$\begin{aligned} \int _0^T (-\lambda _kc_k+F_k) \alpha _k\,dt=\int _0^T c_k(-\lambda _k\alpha _k+G_k)\,dt+u_0^k\alpha '_k(0)-u_1^k\alpha _k(0), \end{aligned}$$

or equivalently

3.28 $$\begin{aligned} \int _0^T F_k \alpha _k\,dt=\int _0^T c_kG_k\,dt+u_0^k\alpha '_k(0)-u_1^k\alpha _k(0). \end{aligned}$$

Summing this identity from $k=1$ to $k=N$ gives

3.29 $$\begin{aligned} \begin{aligned} \int _0^T \big \langle F^{(N)},v_N \big \rangle dt&=\int _0^T \big \langle u_N,G^{(N)}\big \rangle _{L^2(\Omega )}\,dt \\&\quad \, +\big \langle u_0^{(N)},v'_N(0)\big \rangle _{L^2(\Omega )}-\big \langle u_1^{(N)},v_N(0)\big \rangle , \end{aligned} \end{aligned}$$

where we set

$$ F^{(N)}=\sum _{k=1}^N F_k\phi _k,\quad G^{(N)}=\sum _{k=1}^N G_k\phi _k,\quad u_j^{(N)}=\sum _{k=1}^N u_j^k \phi _k $$

for $j=0,1$. That (3.28) and (3.29) are equivalent can be seen by Lemma 3.5. Next, note that $G\in L^2(0,T;\widetilde{L}^2(\Omega ))$ and Lebesgue’s dominated convergence theorem together with Parseval’s identity ensure

$$ \begin{aligned} \big \Vert G^{(N)}(t)\big \Vert _{L^2(\Omega )}^2&=\sum _{k=1}^N \big |\langle G,\phi _k \rangle _{L^2(\Omega )}\big |^2\\&\le \sum _{k=1}^{\infty }\big |\langle G,\phi _k \rangle _{L^2(\Omega )}\big |^2=\Vert G(t)\Vert _{L^2(\Omega )}^2, \end{aligned} $$

which gives

$$ G^{(N)}\rightarrow G\text { in }L^2(0,T;\widetilde{L}^2(\Omega )) $$

as $N\rightarrow \infty$. To see that $F^{(N)}\rightarrow F$ in $L^2(0,T;H^{-s}(\Omega ))$, let us first observe that

3.30 $$\begin{aligned} \begin{aligned} F_k\phi _k&=\left\langle F,\phi _k \right\rangle \phi _k\\&=\langle SF,\phi _k\rangle _{\widetilde{H}^s(\Omega )}\phi _k\\&=\langle SF,S(\lambda _k\phi _k)\rangle _{\widetilde{H}^s(\Omega )}\phi _k\\&=\left\langle F,\lambda _k\phi _k\right\rangle _{H^{-s}(\Omega )}\phi _k\\&=\big \langle F,\lambda _k^{1/2}\phi _k\big \rangle _{H^{-s}(\Omega )}\lambda _k^{1/2}\phi _k. \end{aligned} \end{aligned}$$

As $\big ( \lambda _k^{1/2}\phi _k\big )_{k\in {{\mathbb {N}}}}$ is an orthonormal basis in $H^{-s}(\Omega )$, we deduce from (3.30) that there holds

$$ F^{(N)}(t)\rightarrow F(t)\text { in }H^{-s}(\Omega ) $$

as $N\rightarrow \infty$. Thus, using Lebesgue’s dominated convergence theorem and Parseval’s identity for *G*, we get

$$ F^{(N)}\rightarrow F\text { in }L^2(0,T;H^{-s}(\Omega )) $$

as $N\rightarrow \infty$. So, we can finally pass to the limit in (3.29) to obtain

$$ \int _0^T\langle F(t),v(t)\rangle \,dt=\int _0^T \langle u(t),G(t)\rangle _{L^2(\Omega )}\,dt+\left\langle u_0,v'(0)\right\rangle _{L^2(\Omega )}-\left\langle u_1,v(0)\right\rangle . $$

This establishes that *u* is a solution to (3.5).

*Step 5.* In this final step, we show that the constructed solution *u* is unique. Suppose that $\widetilde{u}$ is another solution, then $U=u-\widetilde{u}$ is a solution to

$$\begin{aligned} {\left\{ \begin{array}{ll} \partial _t^2U +(-\Delta )^sU =0 & \text { in }\Omega _T,\\ U=0 & \text { in }(\Omega _e)_T,\\ U(0)=\partial _t U(0)=0 & \text { in }\Omega . \end{array}\right. } \end{aligned}$$

By Definition 3.1 this means

$$ \int _0^T \langle U(t),G(t)\rangle _{L^2(\Omega )}\,dt=0 $$

for all $G\in L^2(\Omega _T)$, but this clearly implies $U=0$ and hence $u=\widetilde{u}$. $\square$

### Very weak solutions to linear nonlocal wave equations with potential

The purpose of this section is to extend the well-posedness theory of equation (3.5) to linear NWEQs with a nonzero potential.

#### Theorem 3.7

(Well-posedness nonlocal wave equation with potential) Let $F\in L^2(0,T;H^{-s}(\Omega ))$, $u_0\in \widetilde{L}^2(\Omega )$ and $u_1\in H^{-s}(\Omega )$. Furthermore, assume that $q\in L^p(\Omega )$ with *p* satisfying the restriction (1.4). Then the problem

3.31 $$\begin{aligned} {\left\{ \begin{array}{ll} \partial _t^2u +(-\Delta )^su+qu =F & \text { in }\Omega _T,\\ u=0 & \text { in }(\Omega _e)_T,\\ u(0)=u_0, \quad \partial _t u(0)=u_1 & \text { in }\Omega \end{array}\right. } \end{aligned}$$

has a unique very weak solution $u\in C([0,T];\widetilde{L}^2(\Omega ))\cap C^1([0,T];H^{-s}(\Omega ))$.

#### Proof

Let us first note that $qu\in H^{-s}(\Omega )$ for any $u\in L^2(\Omega )$ as

3.32 $$\begin{aligned} \begin{aligned} \bigg |\int _{\Omega }quv\, dx \bigg |&\le \Vert qv\Vert _{L^2(\Omega )}\Vert u\Vert _{L^2(\Omega )}\\&\le \Vert q\Vert _{L^{n/s}(\Omega )}\Vert v\Vert _{L^{\frac{2n}{n-2s}}(\Omega )}\Vert u\Vert _{L^2(\Omega )}\\&\le C\Vert q\Vert _{L^{n/s}(\Omega )}\Vert v\Vert _{\widetilde{H}^s(\Omega )}\Vert u\Vert _{L^2(\Omega )}\\&\le C\Vert q\Vert _{L^{p}(\Omega )}\left\| v \right\| _{\widetilde{H}^s(\Omega )}\Vert u\Vert _{L^2(\Omega )} \end{aligned} \end{aligned}$$

for all $v\in \widetilde{H}^s(\Omega )$. The case $p=\infty$ is clear. In the case $\frac{n}{s}\le p<\infty$ with $2s< n$ we used Hölder’s inequality with

$$ \frac{1}{2}=\frac{n-2s}{2n}+\frac{s}{n}, $$

$L^{r_2}(\Omega )\hookrightarrow L^{r_1}(\Omega )$ for $r_1\le r_2$ as $\Omega \subset {{\mathbb {R}}}^n$ is bounded, and Sobolev’s inequality. In the case $2s>n$ one can use the embedding $H^s({{\mathbb {R}}}^n)\hookrightarrow L^{\infty }({{\mathbb {R}}}^n)$ and the boundedness of $\Omega$ to see that the estimate (3.32) holds. In the case $n=2s$ one can use the boundedness of the embedding $\widetilde{H}^s(\Omega )\hookrightarrow L^{\bar{p}}(\Omega )$ for all $2\le \bar{p}<\infty$, Hölder’s inequality and the boundedness of $\Omega$ to get the final estimate (3.32). The aforementioned embedding in the critical case follows by [48] and the Poincaré inequality. The above clearly implies that for any $u\in C([0,T];\widetilde{L}^2(\Omega ))$, we have $qu\in L^2(0,T;H^{-s}(\Omega ))$ with

3.33 $$\begin{aligned} \Vert qu\Vert _{L^2(0,T;H^{-s}(\Omega ))}\le C\Vert q\Vert _{L^p(\Omega )}\Vert u\Vert _{L^2(\Omega _T)}. \end{aligned}$$

Now, we wish to use a fixed point argument to construct the solution to (3.5).

Via Theorem 3.6, we can define

$$\begin{aligned} \begin{aligned} S:C([0,T];\widetilde{L}^2(\Omega ))&\rightarrow C([0,T];\widetilde{L}^2(\Omega ))\cap C^1([0,T];H^{-s}(\Omega )), \quad v\mapsto u, \end{aligned} \end{aligned}$$

where *u* is the solution of

$$\begin{aligned} {\left\{ \begin{array}{ll} \partial _t^2u +(-\Delta )^su =F-qv & \text { in }\Omega _T,\\ u=0 & \text { in }(\Omega _e)_T,\\ u(0)=u_0, \quad \partial _t u(0)=u_1 & \text { in }\Omega . \end{array}\right. } \end{aligned}$$

Assume that $v_1,v_2\in C([0,T];\widetilde{L}^2(\Omega ))$. Since the function $u=S(v^1)-S(v^2)$ solves

$$ {\left\{ \begin{array}{ll} \partial _t^2u +(-\Delta )^su =-q(v^1-v^2) & \text { in }\Omega _T,\\ u=0 & \text { in }(\Omega _e)_T,\\ u(0)= \partial _t u(0)=0 & \text { in }\Omega , \end{array}\right. } $$

the energy estimate (3.6) (applied for the case $T=t$) yields

$$ \begin{aligned} \Vert u(t)\Vert _{L^2(\Omega )}+\left\| \partial _t u(t)\right\| _{H^{-s}(\Omega )}&\le C\left\| q(v^1-v^2)\right\| _{L^2(0,t;H^{-s}(\Omega ))}, \end{aligned} $$

for a.e. $t\in [0,T]$. Hence, by (3.33) we obtain

3.34 $$\begin{aligned} \Vert u(t)\Vert _{L^2(\Omega )}\le C\Vert q\Vert _{L^p(\Omega )}\left\| v^1-v^2 \right\| _{L^2(\Omega _t)}, \end{aligned}$$

for $t\in [0,T]$. Next, introduce for $\theta >0$ the equivalent norm

$$ \Vert w\Vert _{\theta }{:}= \sup _{0\le t\le T}e^{-\theta t}\Vert w(t)\Vert _{L^2(\Omega )} $$

on $C([0,T];\widetilde{L}^2(\Omega ))$. Then from equation (3.34) we deduce that

$$ \begin{aligned} \Vert u(t)\Vert _{L^2(\Omega )}&\le C\Vert q\Vert _{L^p(\Omega )}\left\| v^1-v^2\right\| _{\theta }\bigg (\int _0^te^{2\theta \tau }\,d\tau \bigg )^{1/2}\\&= \frac{C}{(2\theta )^{1/2}}(e^{2\theta t}-1)^{1/2}\Vert q\Vert _{L^p(\Omega )}\left\| v^1-v^2\right\| _{\theta }\\&\le \frac{C}{(2\theta )^{1/2}}e^{\theta t}\Vert q\Vert _{L^p(\Omega )}\left\| v^1-v^2 \right\| _{\theta } \end{aligned} $$

for $t\in [0,T]$. Dividing by $e^{\theta t}$ and taking the supremum over [0, *T*], this implies

$$ \begin{aligned} \Vert u\Vert _{\theta }&\le \frac{C}{(2\theta )^{1/2}}\Vert q\Vert _{L^p(\Omega )}\left\| v^1-v^2\right\| _{\theta }. \end{aligned} $$

Remembering that $u=S(v^1)-S(v^2)$ and choosing $\theta >0$ such that

$$ C_0{:}= \frac{C}{(2\theta )^{1/2}}\Vert q\Vert _{L^p(\Omega )}<1, $$

we get

$$ \Vert S(v^1)-S(v^2)\Vert _{\theta }\le C_0\Vert v^1-v^2\Vert _{\theta } $$

and thus *S* is a contraction on the complete metric space $(C([0,T];\widetilde{L}^2(\Omega )),\Vert \cdot \Vert _{\theta })$. Therefore, we can apply the Banach fixed point theorem to deduce that *S* has a unique fixed point $u\in C([0,T];\widetilde{L}^2(\Omega ))\cap C^1([0,T];H^{-s}(\Omega ))$. Hence, we have shown the existence of a unique very weak solution, and we can conclude the proof. $\square$

### Properties of very weak solutions

In this section, we establish some relations between various definitions of solutions to linear NWEQs, which can be seen as a consistency test of the introduced notions. In Proposition 3.8 and 3.9, we show that

$$ \text { weak solution }\Rightarrow \text { very weak solution }\Rightarrow \text { distributional solution} $$

and in Corollary 3.10 that

$$ \text { regular very weak solution = weak solution}. $$

#### Proposition 3.8

(Distributional solutions) Let $F\in L^2(0,T;H^{-s}(\Omega ))$, $u_0\in \widetilde{L}^2(\Omega )$, $u_1\in H^{-s}(\Omega )$ and $q\in L^p(\Omega )$ with *p* satisfying the restrictions (1.4). The unique very weak solution of (3.31) is a distributional solution, that is, there holds

3.35 $$\begin{aligned} \int _{\Omega _T}u\left( \partial _t^2\varphi +(-\Delta )^s\varphi +q\varphi \right) dt=\int _0^T\langle F,\varphi \rangle \,dt+\langle u_0,\partial _t\varphi (0)\rangle _{L^2(\Omega )}-\langle u_1,\varphi (0)\rangle , \end{aligned}$$

for all $$\varphi \in C_c^{\infty }([0,T)\times \Omega )$$.

#### Proof

Let us note that it is enough to prove the result for $q=0$ as the general case follows by replacing *F* with $F-qu$.

We use the same notation as in the proof of Theorem 3.6, but this time the $\alpha _k$ are the coefficients in the expansion of $\varphi$ in the orthonormal basis $(\phi _k)_{k\in {{\mathbb {N}}}}$, that is $\alpha _k=\langle \varphi ,\phi _k\rangle _{L^2(\Omega )}$. We start with the identity

$$ \begin{aligned} \int _0^T c''_k \alpha _k\,dt=\int _0^T c_k\alpha ''_k\,dt+u_0^k\alpha '_k(0)-u_1^k\alpha _k(0) \end{aligned} $$

(see (3.27)). Now, using the relation (3.8), we deduce the equality

$$ \int _0^T \left( -\lambda _k c_k+F_k\right) \alpha _k\,dt=\int _0^T c_k\alpha ''_k\,dt+u_0^k\alpha '_k(0)-u_1^k\alpha _k(0). $$

This is equivalent to

3.36 $$\begin{aligned} \int _0^T c_k \left( \alpha ''_k+\lambda _k \alpha _k\right) dt=\int _0^T F_k\alpha _k\,dt+u_1^k\alpha _k(0)-u_0^k\alpha '_k(0). \end{aligned}$$

Next note that

$$ \begin{aligned} \langle (-\Delta )^s\varphi (t),\phi _k\rangle _{L^2(\Omega )}&=\langle (-\Delta )^s\varphi (t),\phi _k\rangle _{L^2({{\mathbb {R}}}^n)}\\&=\langle (-\Delta )^{s/2} \varphi (t),(-\Delta )^{s/2}\phi _k\rangle _{L^2({{\mathbb {R}}}^n)}\\&=\lambda _k\langle \varphi (t),\phi _k\rangle _{L^2(\Omega )}\\&=\lambda _k\alpha _k(t) \end{aligned} $$

for all $0\le t\le T$. Using $\langle \phi _k,\phi _\ell \rangle _{L^2(\Omega )}=\delta _{k\ell }$, we may write

$$ \begin{aligned} \bigg \langle \sum _{k=1}^m c_k\phi _k,\sum _{\ell =1}^m\langle (-\Delta )^s \varphi ,\phi _{\ell }\rangle _{L^2(\Omega )}\phi _{\ell }\bigg \rangle _{L^2(\Omega )}=\sum _{k=1}^m\lambda _kc_k\alpha _k. \end{aligned} $$

Since $u,\chi _{\Omega }(-\Delta )^s\varphi \in L^2(0,T;\widetilde{L}^2(\Omega ))$, where $\chi _\Omega$ is the characteristic function of $\Omega$, the time integral of the left hand side converges to $\langle u,(-\Delta )^s\varphi \rangle _{L^2(\Omega _T)}$. We also have

$$ \int _0^T\bigg \langle \sum _{k=1}^m c_k\phi _k,\sum _{\ell =1}^m\langle \partial _t^2 \varphi ,\phi _{\ell }\rangle _{L^2(\Omega )}\phi _{\ell }\bigg \rangle _{L^2(\Omega )}\,dt=\int _0^T \sum _{k=1}^m c_k\alpha ''_k\,dt\rightarrow \int _{\Omega _T}u\partial _t^2\varphi \,dxdt $$

as $m\rightarrow \infty$. Thus, summing the identity (3.36) from $k=1$ to *m* and passing to the limit $m\rightarrow \infty$ yields

$$ \int _{\Omega _T}u(\partial _t^2\varphi +(-\Delta )^s\varphi )\,dxdt=\int _0^T\langle F,\varphi \rangle \,dt+\langle u_0,\partial _t\varphi (0)\rangle _{L^2(\Omega )}-\langle u_1,\varphi (0)\rangle . $$

For the convergence of the term involving *F* we refer to Step 4 in the proof of Theorem 3.6. Hence, we can conclude the proof. $\square$

#### Proposition 3.9

(Weak solutions are very weak solutions) Suppose that $F\in L^2(0,T;\widetilde{L}^2(\Omega ))$, $u_0\in \widetilde{H}^s(\Omega )$, $u_1\in \widetilde{L}^2(\Omega )$, $q\in L^p(\Omega )$ with *p* satisfying the restrictions (1.4) and $u\in C([0,T];\widetilde{H}^s(\Omega ))\cap C^1([0,T];\widetilde{L}^2(\Omega ))$ is a weak solution of (3.31). Then *u* is a very weak solution of (3.31).

#### Proof

Let us prove the result only in the case $q=0$, as the same proof applies in the general case $q\ne 0$. For the necessary modifications, we refer the reader to [43, Proof of Proposition 4.1].

Let $G\in L^2(0,T;\widetilde{L}^2(\Omega ))$ and suppose $w\in C([0,T];\widetilde{H}^s(\Omega ))\cap C^1([0,T];\widetilde{L}^2(\Omega ))$ is the unique solution to

3.37 $$\begin{aligned} {\left\{ \begin{array}{ll} \partial _t^2w +(-\Delta )^sw =G & \text { in }\Omega _T,\\ w=0 & \text { in }(\Omega _e)_T,\\ w(T)=0, \quad \partial _t w(T)=0 & \text { in }\Omega . \end{array}\right. } \end{aligned}$$

We follow now the proof of [43, Claim 4.2], that is we consider the parabolic regularized problems

$$\begin{aligned} {\left\{ \begin{array}{ll} \partial _t^2u +\varepsilon (-\Delta )^s\partial _t u+(-\Delta )^su = F & \text { in }\Omega _T,\\ u=0 & \text { in }(\Omega _e)_T,\\ u(0)=u_0, \quad \partial _t u(0)=u_1 & \text { in }\Omega \end{array}\right. } \end{aligned}$$

and

$$\begin{aligned} {\left\{ \begin{array}{ll} \partial _t^2w -\varepsilon (-\Delta )^s \partial _t w+(-\Delta )^sw = G& \text { in }\Omega _T,\\ w=0 & \text { in }(\Omega _e)_T,\\ w(T)= \partial _t w(T)=0 & \text { in }\Omega \end{array}\right. } \end{aligned}$$

for $\varepsilon >0$. By [54, Theorem 3.1] or [39, Chapter 3, Theorem 8.3], these regularized problems have a unique (weak) solution

$$\begin{aligned} \begin{aligned} u_{\varepsilon }&\in C([0,T];\widetilde{H}^s(\Omega ))\text { with } {\left\{ \begin{array}{ll} & \hspace{-0.3cm}\partial _t u_{\varepsilon }\in L^2(0,T;\widetilde{H}^s(\Omega ))\cap C([0,T];\widetilde{L}^2(\Omega ))\\ & \hspace{-0.3cm}\partial _t^2 u_{\varepsilon }\in L^2(0,T;H^{-s}(\Omega )) \end{array}\right. } \\ w_{\varepsilon }&\in C([0,T];\widetilde{H}^s(\Omega ))\text { with } {\left\{ \begin{array}{ll} & \hspace{-0.3cm}\partial _t w_{\varepsilon }\in L^2(0,T;\widetilde{H}^s(\Omega ))\cap C([0,T];\widetilde{L}^2(\Omega ))\\ & \hspace{-0.3cm}\partial _t^2 w_{\varepsilon }\in L^2(0,T;H^{-s}(\Omega )) \end{array}\right. } \end{aligned} \end{aligned}$$

and as $\varepsilon \rightarrow 0$, one has

3.38 $$\begin{aligned} \begin{aligned} u_{\varepsilon }\rightarrow u&\text { in }C([0,T];\widetilde{H}^s(\Omega )),\\ \partial _t u_{\varepsilon }\rightarrow \partial _t u&\text { in }C([0,T];\widetilde{L}^2(\Omega )),\\ \partial _t^2u_{\varepsilon }\rightharpoonup \partial _t^2 u&\text { in } L^2(0,T;H^{-s}(\Omega )) \end{aligned} \end{aligned}$$

(cf. [39, Chapter 3, eq. (8.74)]). This ensures that

$$\begin{aligned} \partial _t^2u_{\varepsilon }\overset{*}{\rightharpoonup }\partial _t^2 u \text { in } L^2(0,T;H^{-s}(\Omega )) \text { as }\varepsilon \rightarrow 0. \end{aligned}$$

The convergence results in (3.38) hold for the functions $w_\varepsilon$ and *w* as well. Now, using twice integration by parts, which is allowed by the regularity of the first time derivative of $u_\varepsilon$ and $w_\varepsilon$, we obtain

$$ \int _0^T \left\langle \partial _t^2 u_{\varepsilon },w_\varepsilon \right\rangle \,dt=\int _0^T \left\langle \partial _t^2w_\varepsilon ,u_\varepsilon \right\rangle \,dt-\langle u_1,w_{\varepsilon }(0)\rangle _{L^2(\Omega )}+\langle u_0,\partial _t w_{\varepsilon }(0)\rangle _{\widetilde{H}^s(\Omega )} $$

for any $\varepsilon >0$ (cf. [54, eq. (4.1)]). In this computation, we used the final and initial time conditions for $w_{\varepsilon }$ and $u_{\varepsilon }$, respectively. Now, passing to the limit $\varepsilon \rightarrow 0$ gives

$$ \int _0^T \left\langle \partial _t^2 u,w \right\rangle dt=\int _0^T \left\langle \partial _t^2w,u\right\rangle dt-\langle u_1,w(0)\rangle _{L^2(\Omega )}+\langle u_0,\partial _t w(0)\rangle _{L^2(\Omega )}. $$

By (3.31) and (3.37) this is equivalent to

$$ \int _0^T \langle F,w \rangle \,dt=\int _0^T \left\langle G,u\right\rangle dt-\langle u_1,w(0)\rangle _{L^2(\Omega )}+\langle u_0,\partial _t w(0)\rangle _{L^2(\Omega )}. $$

Hence, we can conclude the proof. $\square$

#### Corollary 3.10

(Regular very weak solutions = weak solutions) Suppose that $F\in L^2(0,T;\widetilde{L}^2(\Omega ))$, $u_0\in \widetilde{H}^s(\Omega )$, $u_1\in \widetilde{L}^2(\Omega )$, $q\in L^p(\Omega )$ with *p* satisfying the restrictions (1.4) and *u* is a very weak solution to (3.31) such that $u\in C([0,T];\widetilde{H}^s(\Omega ))\cap C^1([0,T];\widetilde{L}^2(\Omega ))$. Then *u* is a weak solution of (3.31).

#### Proof

By the usual well-posedness result, the problem (3.31) has a unique weak solution *v*. Using Proposition 3.9, one sees that *v* is a very weak solution to the same problem. By the uniqueness of very weak solutions, it follows that $u=v$, which in turn implies the assertion. $\square$

## Runge approximation and inverse problem for linear NWEQs

As we mentioned in Section 1, a key ingredient to studying nonlocal inverse problems is based on the Runge approximation. In this section, we establish the proof of Theorem 1.2.

### Runge approximation

#### Proof of Theorem 1.2

As usual, we show the Runge approximation property by a Hahn-Banach argument. Hence, we need to show that given $F\in L^2(0,T;H^{-s}(\Omega ))$ vanishing on $\mathscr {R}_W$, it follows that $F=0$.

First observe that if *u* solves (1.6), then $v=u-\varphi$ is the unique solution to

$$\begin{aligned} {\left\{ \begin{array}{ll} \partial _t^2v +(-\Delta )^sv+qv = -(-\Delta )^s\varphi & \text { in }\Omega _T,\\ v=0 & \text { in }(\Omega _e)_T,\\ v(0)=\partial _t v(0)=0 & \text { in }\Omega . \end{array}\right. } \end{aligned}$$

Now, by Theorem 3.7 there is a unique solution *w* of

$$\begin{aligned} {\left\{ \begin{array}{ll} \partial _t^2w +(-\Delta )^sw +qw= F & \text { in }\Omega _T,\\ w=0 & \text { in }(\Omega _e)_T,\\ w(T)= \partial _t w(T)=0 & \text { in }\Omega . \end{array}\right. } \end{aligned}$$

As $\chi _{\Omega }(-\Delta )^s\varphi \in L^2(0,T;\widetilde{L}^2(\Omega ))$, we can use *v* as a test function for the equation of *w* to obtain

$$ -\int _0^T \left\langle w(t),(-\Delta )^s\varphi (t)\right\rangle _{L^2(\Omega )}\,dt=\int _0^T\langle F(t),v(t)\rangle \,dt $$

(see (3.2)). By assumption, the right-hand side vanishes, and hence

$$ \int _0^T\left\langle w(t),(-\Delta )^s\varphi (t)\right\rangle _{L^2(\Omega )}\,dt=0. $$

By taking $\varphi (x,t)=\eta (t)\psi (x)$ with $\eta \in C_c^{\infty }((0,T))$ and $\psi \in C_c^{\infty }(W)$, this implies

$$ (-\Delta )^s w(t)=0\quad \text { for }x\in W\text { and a.e. }t\in [0,T]. $$

As $w\in L^2(0,T;\widetilde{L}^2(\Omega ))$ we know that $w=0$ in $\Omega _e$ and hence the unique continuation principle ensures $w=0$ in ${{\mathbb {R}}}^n$. Now, according to Proposition 3.8, very weak solutions are distributional solutions, and thus we deduce that

$$ \int _0^T\langle F,\Phi \rangle \,dt=0 $$

for all $\Phi \in C_c^{\infty }([0,T)\times \Omega )$ (see (3.35)). This in particular shows that $F=0$ as $C_c^{\infty }(\Omega _T)$ is dense in $L^2(0,T;\widetilde{H}^s(\Omega ))$ and $(L^2(0,T;\widetilde{H}^s(\Omega )))^*=L^2(0,T;H^{-s}(\Omega ))$. This proves the assertion. $\square$

### DN maps for NWEQs

Let us next recall the rigorous definition of the DN map related to NWEQs.

#### Definition 4.1

*(DN map)* Let $\Omega \subset {{\mathbb {R}}}^n$ be a bounded Lipschitz domain, $s>0$ a non-integer, $T>0$ and $q\in L^p(\Omega )$ with *p* satisfying the restrictions (1.4). Then we define the DN map $\Lambda _{q}$ related to

4.1 $$\begin{aligned} {\left\{ \begin{array}{ll} \partial _t^2u +(-\Delta )^su+qu=0 & \text { in }\Omega _T,\\ u=\varphi & \text { in }(\Omega _e)_T,\\ u(0)= \partial _t u(0)=0 & \text { in }\Omega \end{array}\right. } \end{aligned}$$

by

4.2 $$\begin{aligned} \begin{aligned} \left\langle \Lambda _{q}\varphi ,\psi \right\rangle {:}= \int _{{{\mathbb {R}}}^n_T}(-\Delta )^{s/2}u\, (-\Delta )^{s/2}\psi \,dxdt, \end{aligned} \end{aligned}$$

for all $\varphi ,\psi \in C_c^{\infty }((\Omega _e)_T)$, where $u\in C([0,T];H^s({{\mathbb {R}}}^n))\cap C^1([0,T];L^2({{\mathbb {R}}}^n))$ is the unique solution of (4.1) (see [43, Theorem 3.1  & Remark 3.2]).

#### Remark 4.2

Let us mention for our later study of nonlinear NWEQs that if *qu* is replaced by a nonlinear function *f*(*x*, *u*) such that $f:\Omega \times {{\mathbb {R}}}\rightarrow {{\mathbb {R}}}$ satisfies Assumption 1, then we can still define the DN map $\Lambda _f$ by (4.2) for all $\varphi ,\psi \in C_c^{\infty }((\Omega _e)_T)$, where this time $u\in C([0,T];H^s({{\mathbb {R}}}^n))\cap C^1([0,T];L^2({{\mathbb {R}}}^n))$ is the unique solution of DN map $\Lambda _{f}$ related to (1.3) (see [43, Proposition 3.7]).

### Proof of Theorem 1.3

Before giving the proof of Theorem 1.3, let us introduce the following notation

$$\begin{aligned} u^{\star }(x,t)=u(x,T-t) \end{aligned}$$

for the time reversal of the function $u:{{\mathbb {R}}}^n_T\rightarrow {{\mathbb {R}}}$. We first derive a suitable integral identity (cf. e.g. [27, Lemma 2.4] or [54, Lemma 4.3]) and use our improved Runge approximation (Theorem 1.2) to conclude the desired result.

#### Lemma 4.3

(Integral identity) For any $\varphi _1, \varphi _2 \in C^\infty _c((W_1)_T)$, there holds that

4.3 $$\begin{aligned} \left\langle (\Lambda _{q_1}-\Lambda _{q_2})\varphi _1,\varphi _2^{\star }\right\rangle =\int _{\Omega _T} \left( q_1-q_2\right) \left( u_1-\varphi _1\right) \left( u_2-\varphi _2\right) ^{\star }\, dxdt, \end{aligned}$$

where $u_j$ is the unique solution of

$$ {\left\{ \begin{array}{ll} \left( \partial _t^2 +(-\Delta )^s + q_j\right) u = 0 & \text { in }\Omega _T \\ u=\varphi _j & \text { in }(\Omega _e)_T,\\ u(0)= \partial _t u(0)=0 & \text { in }\Omega \end{array}\right. } $$

for $$j=1,2$$.

#### Proof

Let us start by observing that the function $(u_2-\varphi _2)^\star$ is the unique solution of

$$\begin{aligned} {\left\{ \begin{array}{ll} \left( \partial _t^2 +(-\Delta )^s + q_2 \right) u = -(-\Delta )^s\varphi _2^\star & \text { in }\Omega _T \\ u=0 & \text { in }(\Omega _e)_T,\\ u(T)= \partial _t u(T)=0 & \text { in }\Omega \end{array}\right. } \end{aligned}$$

and thus by [43, Claim 4.2] we know that there holds

4.4 $$\begin{aligned} \int _0^T \left\langle \partial _t^2 (u_1-\varphi _1),(u_2-\varphi _2)^\star \right\rangle \,dt=\int _0^T \left\langle \partial _t^2(u_2-\varphi _2)^\star ,(u_1-\varphi _1)\right\rangle dt. \end{aligned}$$

Thus, using the PDEs for $u_1-\varphi _1$ and $(u_2-\varphi _2)^\star$, (4.4) and the symmetry of the fractional Laplacian, we may calculate

$$\begin{aligned} \begin{aligned}&\quad \, \int _{\Omega _T}(q_1-q_2)(u_1-\varphi _1)(u_2-\varphi _2)^\star \,dxdt\\&= -\int _0^T\left\langle (\partial _t^2+(-\Delta )^s)(u_1-\varphi _1),(u_2-\varphi _2)^\star \right\rangle \,dt \\&\quad \, +\int _0^T\left\langle (\partial _t^2+(-\Delta )^s)(u_2-\varphi _2)^\star ,u_1-\varphi _1\right\rangle dt\\&\quad \,-\int _0^T\left\langle (-\Delta )^s\varphi _1,(u_2-\varphi _2)^\star \right\rangle \,dt+\int _0^T\left\langle (-\Delta )^s\varphi _2^\star ,u_1-\varphi _1\right\rangle dt\\&= -\int _0^T\left\langle (-\Delta )^s(u_1-\varphi _1),(u_2-\varphi _2)^\star \right\rangle \,dt+\int _0^T\left\langle (-\Delta )^s(u_2-\varphi _2)^\star ,u_1-\varphi _1\right\rangle dt\\&\quad \,-\int _0^T\left\langle (-\Delta )^s\varphi _1,(u_2-\varphi _2)^\star \right\rangle \,dt+\int _0^T\left\langle (-\Delta )^s\varphi _2^\star ,u_1-\varphi _1\right\rangle dt\\&=-\int _0^T\langle (-\Delta )^s u_2,\varphi _1^\star \rangle \,dt+\int _0^T\langle (-\Delta )^s u_1,\varphi _2^\star \rangle \,dt. \end{aligned} \end{aligned}$$

By definition of the DN map, we deduce that

$$\begin{aligned} \left\langle \Lambda _{q_1}\varphi _1,\varphi _2^\star \right\rangle -\left\langle \Lambda _{q_2}\varphi _2,\varphi _1^\star \right\rangle =\int _{\Omega _T}(q_1-q_2)(u_1-\varphi _1)(u_2-\varphi _2)^\star \,dxdt, \end{aligned}$$

which completes the proof. $\square$

#### Proof of Theorem 1.3

We prove Theorem 1.3 by using the Runge argument. Using Theorem 1.2, we can approximate any $\Psi _j \in C_c^{\infty }(\Omega )$, $j=1,2$, in $L^2(0,T;\widetilde{H}^s(\Omega ))$ by sequences in the Runge sets $\mathscr {R}_{W_1}$ and $\mathscr {R}_{W_2}$, respectively. Since $q_j\in L^p(\Omega )$ satisfies the estimate (3.32), we may pass in (4.3) to the limit and hence taking the condition (1.7) into account, we arrive at

$$ \int _{\Omega _T}(q_1-q_2)\Psi _1\Psi _2^\star \,dxdt=0 $$

for all $\Psi _1,\Psi _2\in C_c^{\infty }(\Omega _T)$. This ensures that $q_1=q_2$ in $\Omega$ as we wanted to show. $\square$

## Well-posedness and inverse problems for nonlinear NWEQs

In this section, we study the inverse problems for NWEQs with polyhomogeneous nonlinearities. We start in Section 5.1 by showing that for any asymptotically polyhomogeneous nonlinearity the expansion is unique. Then, in Section 5.2, by using similar techniques as in [43] and our stronger Runge approximation (Theorem 1.2), we demonstrate Theorem 1.5. Let us note that in the case of asymptotically polyhomogeneous nonlinearities, Theorem 1.5 only shows that the expansion coefficients coincide and not the nonlinearities themselves. This could be improved to $f^{(1)}=f^{(2)}$ in the range $2s>n$ by imposing a suitable decay of the constants $C_N$, $\sum _{k=1}^{N-1}b_k$ as $N\rightarrow \infty$, appearing in Definition 1.4, (ii), but we do not investigate this further in this article.

### Uniqueness of asymptotic expansion

Before discussing the proof of Theorem 1.5, let us make the following observation.

#### Lemma 5.1

Let $\Omega \subset {{\mathbb {R}}}^n$ be a bounded domain. Assume that we have given a sequence $\left( r_k\right) _{k=1}^\infty \subset {{\mathbb {R}}}$ satisfying $0<r_k<r_{k+1}$ for all $k\in {{\mathbb {N}}}$. Let $f:\Omega \times {{\mathbb {R}}}\rightarrow {{\mathbb {R}}}$ be a Carathéodory function and suppose there holds

$$ f\sim \sum _{k\ge 1}f_k \quad \text {and} \quad f\sim \sum _{k\ge 1}\widetilde{f}_k, $$

for two sequences $(f_k)_{k\in {{\mathbb {N}}}}$, $(\widetilde{f}_k)_{k\in {{\mathbb {N}}}}$ satisfying the assumptions in Definition 1.4, (ii) for the same sequence $(r_k)_{k\in {{\mathbb {N}}}}$ but with possibly different constants $C_N$ and $\widetilde{C}_N$ for $N\in {{\mathbb {N}}}_{\ge 2}$. Then we have $f_k=\widetilde{f}_k$ for all $k\in {{\mathbb {N}}}$.

#### Proof

By assumption, we may compute

$$\begin{aligned} \big |f_1(x,1) - {\widetilde{f}}_1(x,1)\big |&= \tau ^{r_1+1}\tau ^{-r_1-1} \big |f_1(x,1) - {\widetilde{f}}_1(x,1)\big |\\&=\tau ^{-r_1-1}\big |f_1(x,\tau ) - {\widetilde{f}}_1(x,\tau )\big |\\&\le \tau ^{-r_1-1}\big (|f(x,\tau ) - {\widetilde{f}}_1(x,\tau )| +|f(x,\tau ) - {\widetilde{f}}_1(x,\tau )|\big )\\&\le \big ( C_2+{\widetilde{C}}_2 \big ) \tau ^{r_2-r_1} \end{aligned}$$

for $x\in \Omega$ and $|\tau |\le 1$. Thus by passing to the limit $\tau \rightarrow 0$, we deduce that $f_1(x,1)={\widetilde{f}}_1(x,1)$ for all $x\in \Omega$ and by homogeneity $f_1={\widetilde{f}}_1$. Continuing inductively, we obtain that $f_k={\widetilde{f}}_k$ for all $k\ge 1$. $\square$

### Recovery of coefficients of polyhomogeneous nonlinearities

Let us start by recalling the following continuity result on Nemytskii operators.

#### Lemma 5.2

(Continuity of Nemytskii operators, [4, Theorem 2.2] and [54, Lemma 3.6]) Let $\Omega \subset {{\mathbb {R}}}^n$ be a bounded domain, $T>0$, and $1\le q,p<\infty$. Assume that $f:\Omega \times {{\mathbb {R}}}\rightarrow {{\mathbb {R}}}$ is a Carathéodory function satisfying

$$\begin{aligned} |f(x,\tau )|\le a+b|\tau |^{\alpha } \end{aligned}$$

for some constants $a,b\ge 0$ and $0<\alpha \le \min (p,q)$. Then the Nemytskii operator *f*, defined by

5.1 $$\begin{aligned} f(u)(x,t){:}= f(x,u(x,t)) \end{aligned}$$

for all measurable functions $u:\Omega _T\rightarrow {{\mathbb {R}}}$, maps continuously $L^p(\Omega )$ to $L^{p/\alpha }(\Omega )$ and $L^q(0,T;L^p(\Omega ))$ into $L^{q/\alpha }(0,T;L^{p/\alpha }(\Omega ))$.

We will use the notation (5.1) introduced in the previous lemma.

#### Proof of Theorem 1.5

Our goal is to show in the first step that the equality of the DN maps for two nonlinearities $f^{(1)}$ and $f^{(2)}$ implies $f^{(1)}(x,v)=f^{(2)}(x,v)$ for any solution to a linear wave equation (see (5.3)). Then in a second step, we use the Runge approximation property of the solutions *v* in $L^2(0,T; \widetilde{H}^s(\Omega ))$ to deduce that all expansion coefficients $f^{(1)}_k$, $f^{(2)}_k$ for $k\in {{\mathbb {N}}}$ agree. In the following, we will often abbreviate $f(x,u)={:}f(u)$.

Let $\varepsilon >0$. We start by observing that for $j=1,2$ the unique (weak) solution $u_\varepsilon ^{(j)}$ of

5.2 $$\begin{aligned} {\left\{ \begin{array}{ll} \partial _t^2u +(-\Delta )^su+f^{(j)}(u)=0 & \text {in }\Omega _T,\\ u=\varepsilon \varphi & \text {in }(\Omega _e)_T,\\ u(0)= \partial _t u(0)=0 & \text {in }\Omega , \end{array}\right. } \end{aligned}$$

can be expanded as $u^{(j)}_\varepsilon = \varepsilon v+ R^{(j)}_\varepsilon$, where *v* and $R^{(j)}_{\varepsilon }$ solve

5.3 $$\begin{aligned} {\left\{ \begin{array}{ll} \partial _t^2v +(-\Delta )^sv=0 & \text {in }\Omega _T,\\ v=\varphi & \text {in }(\Omega _e)_T,\\ v(0)= \partial _t v(0)=0 & \text {in }\Omega \end{array}\right. } \end{aligned}$$

and

$$\begin{aligned} {\left\{ \begin{array}{ll} \partial _t^2R +(-\Delta )^s R= -f^{(j)}\big (u^{(j)}_\varepsilon \big ) & \text {in }\Omega _T,\\ R=0 & \text {in }(\Omega _e)_T,\\ R(0)= \partial _t R(0)=0 & \text {in }\Omega , \end{array}\right. } \end{aligned}$$

respectively. By (1.11), the UCP for the fractional Laplacian and (5.2) guarantee

5.4 $$\begin{aligned} u_{\varepsilon }{:}= u_{\varepsilon }^{(1)}=u_{\varepsilon }^{(2)},\,R_{\varepsilon }{:}= R_{\varepsilon }^{(1)}=R_{\varepsilon }^{(2)} \text { and } f^{(1)}(u_\varepsilon )=f^{(2)}(u_\varepsilon ). \end{aligned}$$

Note that we have $f^{(j)}(x,0)=0$ for a.e. $x\in \Omega$ and for $j=1,2$. For serially polyhomogeneous nonlinearities, this follows from the pointwise convergence (1.8) and the homogeneity of the expansion coefficients $f_k^{(j)}$ for $k\in {{\mathbb {N}}}$ and $j=1,2$. In contrast, for asymptotically polyhomogeneous nonlinearities it is a consequence of (1.10) and again the homogeneity of the functions $f_k^{(j)}$. Next, we claim that we have

5.5 $$\begin{aligned} |f^{(j)}(x,\tau )| \lesssim |\tau |^{r_1+1}+|\tau |^{r_{\infty }+1} \end{aligned}$$

for a.e. $x\in \Omega$ and all $\tau \in {{\mathbb {R}}}$. First, suppose that $f^{(j)}$ is serially polyhomogeneous. Notice that (1.12) and the ratio test imply the convergence

5.6 $$\begin{aligned} 0\le \sum _{k\ge 1}b^{(j)}_k<\infty \end{aligned}$$

and hence, using (1.9) and the Weierstrass M-test, we can conclude that

$$\begin{aligned} \sum _{k\ge 1}f^{(j)}_k\text { converges absolutely and uniformly for a.e. }x\in \Omega \text { and all }|\tau |\le 1. \end{aligned}$$

On the one hand, for $|\tau |\le 1$, we use (1.9) and (5.6) to derive the following estimate

5.7 $$\begin{aligned} |f^{(j)}(x,\tau )|\le \sum _{k\ge 1}|f^{(j)}_k(x,\tau )| \le \sum _{k\ge 1}b^{(j)}_k|\tau |^{r_k+1}\le |\tau |^{r_1+1}\sum _{k\ge 1}b_k^{(j)}. \end{aligned}$$

On the other hand, for $|\tau |>1$, we use the pointwise convergence (1.8), the homogeneity of $f_k$ and (5.7) to get

5.8 $$\begin{aligned} \begin{aligned} |f^{(j)}(x,\tau )|&=\bigg |\sum _{k\ge 1}f^{(j)}_k(x,\tau )\bigg |=\bigg |\sum _{k\ge 1}|\tau |^{r_k+1}f^{(j)}_k(x,\tau /|\tau |)\bigg |\\&\le |\tau |^{r_{\infty }+1}\sum _{k\ge 1}|f^{(j)}_k(x,\tau /|\tau |)|\\&\le |\tau |^{r_{\infty }+1} \sup _{\sigma =\pm 1}\sum _{k\ge 1}|f^{(j)}_k(x,\sigma )|\\&\le |\tau |^{r_{\infty }+1} \sum _{k\ge 1}b_k^{(j)}. \end{aligned} \end{aligned}$$

So, combining (5.7) and (5.8) shows that (5.5) holds for serially polyhomogeneous nonlinearities up to a constant, which is given by $\sum _{k\ge 1} b_k^{(j)}$, for $j=1,2$.

Next, assume that $f^{(j)}$ is asymptotically polyhomogeneous. In this case, (1.10) demonstrates that for $|\tau |\le 1$

$$\begin{aligned} \begin{aligned} |f^{(j)}(x,\tau )|&\le |f^{(j)}(x,\tau )-f^{(j)}_1(x,\tau )|+|f^{(j)}_1(x,\tau )|\\&\le C_2 |\tau |^{r_2+1}+ b_1|\tau |^{r_1+1} \\&\lesssim |\tau |^{r_1+1}, \end{aligned} \end{aligned}$$

showing that the estimate (5.5) also holds with asymptotically polyhomogeneous case for a.e. $x\in \Omega$ and all $\tau \in {{\mathbb {R}}}$. Hence, (5.5) also holds in this case.

Continuing as in [43, Proof of Theorem 1.1], we obtain the estimates

1. $$\left\| f^{(j)}(\cdot ,u_{\varepsilon })\right\| _{L^2(\Omega _T)} \lesssim \Vert u_\varepsilon \Vert _{L^\infty (0,T;H^s({{\mathbb {R}}}^n))}^{r_1+1} + \Vert u_\varepsilon \Vert _{L^\infty (0,T;H^s({{\mathbb {R}}}^n))}^{r_\infty +1}$$,
2. $$ \Vert R_\varepsilon \Vert _{L^\infty (0,T;\widetilde{H}^s(\Omega ))} + \Vert \partial _t R_\varepsilon \Vert _{L^\infty (0,T; L^2(\Omega ))}$$ $$\lesssim \Vert u_\varepsilon \Vert _{L^\infty (0,T;H^s({{\mathbb {R}}}^n))}^{r_1+1} + \Vert u_\varepsilon \Vert _{L^\infty (0,T;H^s({{\mathbb {R}}}^n))}^{r_\infty +1}$$
3. $$ \Vert u_\varepsilon \Vert _{L^{\infty }(0,T; H^s({{\mathbb {R}}}^n))} + \Vert \partial _t u_\varepsilon \Vert _{L^\infty (0,T; L^2({{\mathbb {R}}}^n))}\lesssim \varepsilon $$.

Therefore, we deduce that

5.9 $$\begin{aligned} \begin{aligned}&\quad \, \Vert R_\varepsilon \Vert _{L^\infty (0,T;\widetilde{H}^s(\Omega ))} + \Vert \partial _t R_\varepsilon \Vert _{L^\infty (0,T; L^2(\Omega ))}\lesssim \varepsilon ^{r_1+1}+\varepsilon ^{r_\infty +1}. \end{aligned} \end{aligned}$$

Now, (5.4) implies

5.10 $$\begin{aligned} f^{(1)}(\varepsilon v + R_\varepsilon ) = f^{(2)}(\varepsilon v+R_\varepsilon ). \end{aligned}$$

Our next goal is to determine iteratively the terms in the series

$$ \sum _{k\ge 1} f^{(j)}_k(\tau ),\quad j=1,2, $$

starting from $f_1^{(j)}$. We distinguish two cases $2s<n$ and $2s\ge n$ as follows:

**Case** $$2s<n$$.

As $r_\infty$ satisfies $0<r_\infty \le \frac{2s}{n-2s}$, we have

5.11 $$\begin{aligned} 1<1+r_k<1+r_\infty \le \frac{n}{n-2s}<\frac{2n}{n-2s}={:}p \end{aligned}$$

for all $k\in {{\mathbb {N}}}$. Using (5.9) and the Sobolev embedding, we see that

5.12 $$\begin{aligned} \varepsilon ^{-1}R_{\varepsilon }\rightarrow 0\quad \text {in}\quad L^q(0,T;L^{p}(\Omega )) \end{aligned}$$

as $\varepsilon \rightarrow 0$ for any $1\le q \le \infty$. Through the estimate

$$\begin{aligned} |f^{(j)}(x,\tau )| \le A^{(j)} + B^{(j)}|\tau |^{r_\infty +1}, \end{aligned}$$

for appropriate constants $A^{(j)},B^{(j)}\ge 0$ (see Assumption 1 or (5.5)), and (1.9), we infer from Lemma  5.2 and (5.11) that the mappings

$$\begin{aligned}&f^{(j)}:L^q(0,T; L^p(\Omega )) \rightarrow L^\frac{q}{r_\infty +1}(0,T; L^\frac{p}{r_\infty +1}(\Omega )),\\&f^{(j)}_k:L^q(0,T; L^p(\Omega )) \rightarrow L^\frac{q}{r_k+1}(0,T; L^\frac{p}{r_k+1}(\Omega )),\quad k\in {{\mathbb {N}}}, \end{aligned}$$

are continuous as long as *q* is chosen such that $q\ge r_\infty +1$ and $q\ge r_k+1$, respectively.

Next, we use the inequalities $r_k< r_\ell \le r_{\infty }$, $k\le \ell$, and the boundedness of $\Omega$ to deduce the inclusion

$$\begin{aligned} L^\frac{q}{r_k+1}(0,T; L^\frac{p}{r_k+1}(\Omega )) \hookrightarrow L^\frac{q}{r_\ell +1}(0,T; L^\frac{p}{r_\ell +1}(\Omega )), \end{aligned}$$

which ensures that the map

5.13 $$\begin{aligned} f^{(j)}_k:L^q(0,T; L^p(\Omega )) \rightarrow L^\frac{q}{r_\ell +1}(0,T; L^\frac{p}{r_\ell +1}(\Omega )),\quad 0<k\le \ell \le \infty \end{aligned}$$

is continuous.

**Serially polyhomogeneous nonlinearity for** $\mathbf {k=1}$: Multiplying (5.10) by $\varepsilon ^{-r_1-1}$ and using the homogeneity of $f_k^{(j)}$, we have pointwise the identity

5.14 $$\begin{aligned} \varepsilon ^{-r_1-1} f^{(j)}(u_\varepsilon ) = \sum _{k=1}^\infty f_k^{(j)}(\varepsilon ^{-\frac{r_1+1}{r_k+1}} u_\varepsilon ),\quad j=1,2, \end{aligned}$$

where

$$\begin{aligned} {\left\{ \begin{array}{ll} -\frac{r_1+1}{r_k+1} = -1, &  \text {if }k=1,\\ -\frac{r_1+1}{r_k+1} >-1, &  \text {if }k\ge 2. \end{array}\right. } \end{aligned}$$

Let us denote by $C_S>0$ the optimal Sobolev constant for the embedding $H^s({{\mathbb {R}}}^n)\hookrightarrow L^p({{\mathbb {R}}}^n)$ and by $D>0$ the constant in the estimate (E3). Furthermore, recall that we have

5.15 $$\begin{aligned} \big |f^{(j)}_k(x,\tau )\big |\le b^{(j)}_k|\tau |^{r_k+1}, \quad r_k<r_{k+1}\le r_{\infty } \end{aligned}$$

for $j=1,2$ (see (1.9)). Also note that $p=2n/(n-2s)$ (see (5.11)) and sufficiently large *q* satisfy

$$ 1\le \frac{p}{r_\infty -r_k}<\infty ,\,1\le \frac{q}{r_\infty -r_k}<\infty $$

(cf., e.g., (5.11)). Hence, we may decompose

$$\begin{aligned} \frac{r_\infty +1}{p}=\frac{r_k+1}{p}+\frac{r_\infty -r_k}{p}\text { and }\frac{r_\infty +1}{q}=\frac{r_k+1}{q}+\frac{r_\infty -r_k}{q}. \end{aligned}$$

Thus, for $0<\varepsilon \le 1$ and $k\ge 1$, we may compute

5.16 $$\begin{aligned} \begin{aligned}&\big \Vert f_k^{(j)}\big (\varepsilon ^{-\frac{r_1+1}{r_k+1}}u_\varepsilon \big )\big \Vert _{L^{\frac{q}{r_\infty + 1}}L^{\frac{p}{r_\infty +1}}}\\&\quad =\varepsilon ^{-(r_1+1)} \big \Vert f_k^{(j)}(u_\varepsilon )\big \Vert _{L^{\frac{q}{r_\infty + 1}}L^{\frac{p}{r_\infty +1}}}\\&\quad \le \underbrace{\varepsilon ^{-(r_1+1)}|\Omega |^{\frac{r_\infty -r_k}{p}}T^{\frac{r_\infty - r_k}{q}}\Vert f_k^{(j)}(u_\varepsilon )\Vert _{L^{\frac{q}{r_k+1}} L^{\frac{p}{r_k+1}}}}_ {\text {by H}\ddot{{\textrm{o}}}\text {lder's inequality}}\\&\quad \le \underbrace{\varepsilon ^{-(r_1+1)}|\Omega |^{\frac{r_\infty -r_k}{p}}T^{\frac{r_\infty - r_k}{q}}b_k^{(j)}\Vert u_\varepsilon \Vert _{L^q L^p}^{r_k+1}}_{\text {by} (\text {5.15})}\\&\quad \le \varepsilon ^{-(r_1+1)}|\Omega |^{\frac{r_\infty -r_k}{p}}T^{\frac{r_\infty +1}{q}}b_k^{(j)}\Vert u_\varepsilon \Vert _{L^{\infty } L^p}^{r_k+1}\\&\quad \le \underbrace{\varepsilon ^{-(r_1+1)}C_s^{r_k+1}|\Omega |^{\frac{r_\infty -r_k}{p}}T^{\frac{r_\infty +1}{q}}b_k^{(j)}\Vert u_\varepsilon \Vert _{L^{\infty } H^s}^{r_k+1}}_ {\text {by Sobolev's inequality}}\\&\quad \le \underbrace{\varepsilon ^{r_k-r_1}(C_sD)^{r_k+1}|\Omega |^{\frac{r_\infty -r_k}{p}}T^{\frac{r_\infty +1}{q}}b_k^{(j)}}_ {\text {by } (\text {E3})}\\&\quad \le \varepsilon ^{r_k-r_1}(\max (1,C_sD))^{r_k+1}(\max (1,|\Omega |))^{\frac{r_\infty -r_k}{p}}T^{\frac{r_\infty +1}{q}}b_k^{(j)}\\&\quad \le \underbrace{(\max (1,C_sD))^{r_\infty +1}(\max (1,|\Omega |))^{\frac{r_\infty }{p}}T^{\frac{r_\infty +1}{q}}b_k^{(j)}}_ {\text {by }\varepsilon \le 1}\\&\quad ={:}M_k^{(j)}, \end{aligned} \end{aligned}$$

for $k\in {{\mathbb {N}}}$ and $j=1,2$, where we abbreviated $L^{\alpha }(0,T;X(U))$ as $L^{\alpha }X$ for any Banach space *X*(*U*) over a spatial domain $U\subset {{\mathbb {R}}}^n$ and $1\le {\alpha }\le \infty$. Since the constants in $M_k^{(j)}$ in front of $b_k^{(j)}$ are independent of *k*, we can use (1.12) and the ratio test to get

$$\begin{aligned} \sum _{k\ge 1}M_k^{(j)}<\infty . \end{aligned}$$

Thus, the Weierstrass M-test ensures that

5.17 $$\begin{aligned} \sum _{k\ge 1}f_k^{(j)}\big (\varepsilon ^{-\frac{r_1+1}{r_k+1}}u_\varepsilon \big ) \text { converges uniformly and absolutely in }L^{\frac{q}{r_\infty + 1}}L^{\frac{p}{r_\infty +1}}, \end{aligned}$$

for $0<\varepsilon \le 1$. By Tannery’s theorem^2^ [41], this is essentially a consequence of Lebesgue’s dominated convergence theorem, (5.14), and the fact that

$$\begin{aligned} f_k^{(j)}\big (\varepsilon ^{-\frac{r_1+1}{r_k+1}}u_\varepsilon \big )\rightarrow {\left\{ \begin{array}{ll} 0, & \text { for }k\ge 2\\ f^{(j)}(v),& \text { for }k=1 \end{array}\right. } \quad \text {as }\varepsilon \rightarrow 0 \end{aligned}$$

(see (5.16) for $k\ge 2$ and (5.12), (5.13) for $k=1$), we may deduce that

$$\begin{aligned} \lim _{\varepsilon \rightarrow 0}\varepsilon ^{-r_1-1} f^{(j)}(u_\varepsilon )=\sum _{k\ge 1}\lim _{\varepsilon \rightarrow 0}f_k^{(j)}\big (\varepsilon ^{-\frac{r_1+1}{r_k+1}} u_\varepsilon \big )=f_1^{(j)}(v) \end{aligned}$$

in $L^{\frac{q}{r_\infty +1}}(0,T;L^{\frac{p}{r_\infty + 1}}(\Omega ))$. Finally, combining this with (5.10) yields

5.18 $$\begin{aligned} f_1^{(1)}(v)=f_1^{(2)}(v) \end{aligned}$$

in $$L^\frac{q}{r_\infty +1}(0,T; L^\frac{p}{r_\infty +1}(\Omega ))$$.

**Asymptotically polyhomogeneous nonlinearity for** $\mathbf {k=1}$: In this case, using Definition 1.4 (ii), we get for $N=2$ that

$$ \big | f^{(j)}(x,\tau ) - f_1^{(j)}(x,\tau ) \big |\le C_2 \left| \tau \right| ^{r_2+1} $$

when $$|\tau |\le 1$$ and

$$\begin{aligned} \big | f^{(j)}(x,\tau ) - f_1^{(j)}(x,\tau ) \big |\le \big |f^{(j)}(x,\tau )|+|f_1^{(j)}(x,\tau )\big | \lesssim |\tau |^{r_\infty +1} \end{aligned}$$

when $|\tau |>1$ for a.e. $x\in \Omega$, yielding

5.19 $$\begin{aligned} \big | f^{(j)}(u_\varepsilon ) - f_1^{(j)}(u_\varepsilon ) \big |\lesssim \left| u_\varepsilon \right| ^{r_2+1} + \left| u_\varepsilon \right| ^{r_\infty +1}. \end{aligned}$$

Above the term $|u_\varepsilon |^{r_2+1}$ can be estimated in $L^\frac{q}{r_\infty +1}L^\frac{q}{r_\infty +1}$ using (5.16) as

$$ \big \Vert |u_\varepsilon |^{r_2+1}\big \Vert _{L^{\frac{q}{r_\infty + 1}}L^{\frac{p}{r_\infty +1}}} \lesssim \Vert u_\varepsilon \Vert ^{r_2+1}_{L^\infty H^s}. $$

The latter term of (5.19) can be directly estimated as

$$\begin{aligned} \Vert |u_\varepsilon |^{r_\infty +1}\Vert _{L^{\frac{q}{r_\infty + 1}}L^{\frac{p}{r_\infty +1}}} \le \Vert u_\varepsilon \Vert _{L^qL^p}^{r_\infty +1} \lesssim \Vert u_\varepsilon \Vert _{L^\infty H^s}^{r_\infty +1}. \end{aligned}$$

Multiplying the above inequality by $\varepsilon ^{-r_1-1}$ with $0<\varepsilon <1$, recalling $r_2>r_1$, and using (E3) we get in the $L^\frac{q}{r_{\infty }+1}L^\frac{p}{r_{\infty }+1}$-norm

5.20 $$\begin{aligned} \begin{aligned}&\big \Vert \varepsilon ^{-r_1-1} f^{(j)}(u_{\varepsilon }) - f_1^{(j)}(\varepsilon ^{-1} u_{\varepsilon })\big \Vert _{L^\frac{q}{r_{\infty }+1}L^\frac{p}{r_{\infty }+1}} \lesssim \varepsilon ^{r_2-r_1}+\varepsilon ^{r_\infty -r_1}\rightarrow 0 \end{aligned} \end{aligned}$$

as $\varepsilon \rightarrow 0$. Therefore, using (5.13) and (5.20), we deduce that

$$\begin{aligned} f_1^{(j)}(v) = \lim _{\varepsilon \rightarrow 0} f_1^{(j)}(\varepsilon ^{-1}u_\varepsilon ) = \lim _{\varepsilon \rightarrow 0} \varepsilon ^{-r_1-1} f^{(j)}(u_\varepsilon ) \end{aligned}$$

in $L^\frac{q}{r_{\infty }+1}(0,T;L^\frac{p}{r_{\infty }+1}(\Omega ))$. So, taking into account (5.10), we get

5.21 $$\begin{aligned} f_1^{(1)}(v)=f_1^{(2)}(v) \end{aligned}$$

in $$L^\frac{q}{r_{\infty }+1}(0,T; L^\frac{p}{r_{\infty }+1}(\Omega ))$$.

Next, let $\Psi \in C_c^{\infty }(\Omega _T)$. By Theorem 1.2, there exists a sequence $(\psi _k)_{k\in {{\mathbb {N}}}}\subset C_c^{\infty }((W_1)_T)$ such that the unique solutions $(v_k)_{k\in {{\mathbb {N}}}}$ of

$$ {\left\{ \begin{array}{ll} \partial _t^2v_k +(-\Delta )^sv_k=0 & \text {in }\Omega _T,\\ v_k=\psi _k & \text {in }(\Omega _e)_T,\\ v_k(0)= \partial _t v_k(0)=0 & \text {in }\Omega \end{array}\right. } $$

satisfy $v_k-\psi _k\rightarrow \Psi$ in $L^2(0,T;\widetilde{H}^s(\Omega ))$ as $k\rightarrow \infty$. Up to extracting a subsequence, we have, by Sobolev’s embedding theorem, that there holds

$$ v_k(t)\rightarrow \Psi (t)\quad \text {in}\quad L^{p}(\Omega ) $$

for a.e. $t\in [0,T]$. Hence, by Lemma 5.2, Hölder’s inequality, and (5.18) or (5.21), we get

$$ f_1^{(1)}(\Psi (t))=\lim _{k\rightarrow \infty }f_1^{(1)}(v_k(t))=\lim _{k\rightarrow \infty }f_1^{(2)}(v_k(t))=f_1^{(2)}(\Psi (t)) $$

in $L^{\frac{p}{r_\infty +1}}(\Omega )$ (or $L^{\frac{p}{r_2+1}}(\Omega )$) for a.e. $t\in [0,T]$. As $f_1^{(j)}$ are Carathéodory functions this needs to hold for all $t\in [0,T]$ and hence

$$ f_1^{(1)}(x,\Psi (x,t))=f_1^{(2)}(x,\Psi (x,t)) $$

for all $$(x,t)\in \Omega _T$$.

Now, let us fix $t_0\in (0,T)$ and $x_0\in \Omega$. Then we choose $\Psi (x,t)=\eta (t)\Phi (x)$ with $\eta \in C_c^{\infty }((0,T))$ and $\Phi \in C_c^{\infty }(\Omega )$, where $\eta ,\Phi$ satisfy $\eta (t)=1$ in a neighborhood of $t_0$ and $\Phi (x)=1$ in a neighborhood of $x_0$. Therefore, evaluating the previous relation at $t=t_0$ we obtain

$$ f_1^{(1)}(x,\Phi (x))=f_1^{(2)}(x,\Phi (x)) $$

for a.e. $x\in \Omega$. This gives $f_1^{(1)}(x_0,1)=f_1^{(2)}(x_0,1)$. Now, the homogeneity assumptions on $f_k^{(j)}$ ensures that $f_1^{(1)}(x,\rho )=f_1^{(2)}(x,\rho )$ for all $x\in \Omega$ and $\rho \in {{\mathbb {R}}}$.

Using a similar approach, one can inductively recover the higher-order terms. In fact, one can argue as follows.

**Serially polyhomogeneous nonlinearity for** $\mathbf {k\ge 2}$: We start by introducing the function

$$ f^{(j),2}{:}= f^{(j)}-f^{(1)}_1= \sum _{k\ge 2}f_k^{(j)} $$

for $j=1,2$ and we observe that $f^{(j),2}$ is again Carathéodory function satisfying the same properties as $f^{(j)}$ itself. Then, $f^{(1)}_1=f^{(2)}_1$ and (5.4) imply that

$$ f^{(1),2}(u_\varepsilon )=f^{(2),2}(u_\varepsilon ). $$

Repeating the same proof as above, but this time multiplying with $\varepsilon ^{-r_2-1}$, we deduce $f^{(1)}_2=f^{(2)}_2$. Thus, iteratively, we get $f^{(1)}_k=f^{(2)}_k$ for any $k\in {{\mathbb {N}}}$.

**Asymptotically polyhomogeneous nonlinearity for** $\mathbf {k\ge 2}$: Using $f^{(1)}_1=f^{(2)}_1$, (1.10) for $N=3$, Sobolev’s embedding and (E3), we get

$$ \begin{aligned}&\Vert (\varepsilon ^{-r_2-1}f^{(1)}(u_\varepsilon )-f_2^{(1)}(\varepsilon ^{-1}u_\varepsilon ))-(\varepsilon ^{-r_2-1}f^{(2)}(u_\varepsilon )-f_2^{(2)}(\varepsilon ^{-1}u_\varepsilon ))\Vert _{L^{\frac{q}{r_{\infty }+1}}L^{\frac{p}{r_{\infty }+1}}}\\&\quad =\varepsilon ^{-r_2-1}\Vert (f^{(1)}(u_\varepsilon )-f_2^{(1)}(u_\varepsilon ))-(f^{(2)}(u_\varepsilon )-f_2^{(2)}(u_\varepsilon ))\Vert _{L^{\frac{q}{r_{\infty }+1}}L^{\frac{p}{r_{\infty }+1}}}\\&\quad =\varepsilon ^{-r_2-1}\Vert (f^{(1)}(u_\varepsilon )-f_2^{(1)}(u_\varepsilon )-f_1^{(1)}(u_\varepsilon ))-(f^{(2)}(u_\varepsilon )-f_2^{(2)}(u_\varepsilon )-f_1^{(2)}(u_\varepsilon ))\Vert _{L^{\frac{q}{r_{\infty }+1}}L^{\frac{p}{r_{\infty }+1}}}\\&\quad \le \varepsilon ^{-r_2-1}\sum _{j=1,2}\Vert f^{(j)}(u_\varepsilon )-f^{(j)}_2(u_\varepsilon )-f_1^{(j)}(u_\varepsilon )\Vert _{L^{\frac{q}{r_{\infty }+1}}L^{\frac{p}{r_{\infty }+1}}}\\&\quad \lesssim \varepsilon ^{-r_2-1}\Vert u_\varepsilon \Vert ^{r_3+1}_{L^{q}L^{p}}\\&\quad \lesssim \varepsilon ^{r_3-r_2}\rightarrow 0 \end{aligned} $$

as $\varepsilon \rightarrow 0$. Taking into account (5.13), this guarantees

$$ \begin{aligned} f_2^{(1)}(v)-f_2^{(2)}(v)&=\lim _{\varepsilon \rightarrow 0}(f_2^{(1)}(\varepsilon ^{-1}u_\varepsilon )-f_2^{(2)}(\varepsilon ^{-1}u_\varepsilon ))\\&=\lim _{\varepsilon \rightarrow 0}\varepsilon ^{-r_2-1}(f^{(1)}(u_\varepsilon )-f^{(2)}(u_\varepsilon ))=0 \end{aligned} $$

in $L^{\frac{q}{r_{\infty }+1}}L^{\frac{p}{r_{\infty }+1}}$. Now, one can repeat the above argument to find that $f_2^{(1)}=f_2^{(2)}$. Therefore, we iteratively get $f_k^{(1)}=f_k^{(2)}$ for all $k\in {{\mathbb {N}}}$.

**Case** $$2s\ge n$$.

The proof is almost the same as the Sobolev embedding guarantees that we have $H^s({{\mathbb {R}}}^n)\hookrightarrow L^p({{\mathbb {R}}}^n)$ for any $2\le p<\infty$ (see [48] for the critical case $2s=n$). Moreover, let us note that in the supercritical case $2s>n$, we only need (1.10) for $|\tau |\le 1$ by the Sobolev embedding $H^s({{\mathbb {R}}}^n)\hookrightarrow L^{\infty }({{\mathbb {R}}}^n)$ and the estimate (E3).

Hence, we have shown that the expansion coefficients of the nonlinearities $f^{(j)}$, $j=1,2$, coincide in both cases and we can conclude the proof. $\square$

## Data Availability

No datasets were generated or analyzed during the current study.

## Proof of Lemma 3.5

In this appendix, we provide the proof of the spectral theoretic lemma, Lemma 3.5. We again denote by $\langle \cdot ,\cdot \rangle$ the duality pairing between $\widetilde{H}^s(\Omega )$ and $H^{-s}(\Omega )$,

where the spaces $\widetilde{H}^s(\Omega )$, $H^{-s}(\Omega )$ are endowed with the norms $\Vert \cdot \Vert _{\widetilde{H}^s(\Omega )}$ and $\Vert \cdot \Vert _{H^{-s}(\Omega )}$ as before.

### Proof of Lemma 3.5

We start by constructing the sequence of eigenvalues $(\lambda _k)_{k\in {{\mathbb {N}}}}$.

Let $L:\widetilde{L}^2(\Omega )\rightarrow \widetilde{L}^2(\Omega )$ be the compact self-adjoint operator given by $L=K\circ S$, where

$$\begin{aligned} \begin{aligned} S:\widetilde{L}^2(\Omega )\rightarrow \widetilde{H}^s(\Omega ), \quad F\mapsto u \end{aligned} \end{aligned}$$

is the source-to-solution map of the problem

$$ {\left\{ \begin{array}{ll} (-\Delta )^su =F & \text { in }\Omega ,\\ u=0 & \text { in }\Omega _e \end{array}\right. } $$

and $K:\widetilde{H}^s(\Omega )\rightarrow \widetilde{L}^2(\Omega )$ denotes the usual inclusion, which is compact by the Rellich-Kondrachov theorem. Note that the solution map *S* is well-defined and continuous by the Lax-Milgram theorem. Hence, it is clear that *L* is compact. The operator *L* is also self-adjoint, because the related bilinear form to $(-\Delta )^s$ is symmetric. Furthermore, if $F\in \widetilde{L}^2(\Omega )$ and $u=LF$, then we have

$$\begin{aligned} \langle LF,F\rangle _{L^2(\Omega )}=\langle u,F\rangle _{L^2(\Omega )}=\big \langle (-\Delta )^{s/2}u,(-\Delta )^{s/2} u \big \rangle _{L^2({{\mathbb {R}}}^n)}=\Vert u\Vert _{\widetilde{H}^s(\Omega )}^2\ge 0. \end{aligned}$$

If $F\ne 0$, then we have $\langle LF,F\rangle _{L^2(\Omega )}>0$ as otherwise *u* would vanish and hence $F=0$. Therefore, *L* is positive definite with $\ker L=\{0\}$. By the spectral theory for compact self-adjoint operators, we deduce that $\sigma (L)=\sigma _p(L)\subset {{\mathbb {R}}}_+$ is at most countable with accumulation point $\mu =0$. Here, $\sigma _p(L)$ denotes the point spectrum of *L*, that is, the set of eigenvalues. Moreover, for any $\mu \in \sigma _p(L)$ its related eigenspace $\text {ker}(L-\mu )$ is finite dimensional. Next, observe that $\mu >0$ is an eigenvalue of *L* if and only if $\lambda =1/\mu$ is an eigenvalue for $(-\Delta )^s$ and $F\in \widetilde{L}^2(\Omega )$ is an eigenfunction of *L* with eigenvalue $\mu >0$ if and only if $u{:}=SF\in \widetilde{H}^s(\Omega )$ is an eigenfunction of $(-\Delta )^s$ with eigenvalue $\lambda =1/\mu$. Therefore, we may conclude that $\sigma _p((-\Delta )^s)$ is an unbounded countable sequence and the corresponding eigenspaces are finite-dimensional.

*Step 1. The first eigenvalue.*

Let us define

$$\begin{aligned} \lambda _1=\inf \left\{ \Vert u\Vert _{\widetilde{H}^s(\Omega )}^2\,;\, u\in \widetilde{H}^s(\Omega ),\,\Vert u\Vert _{L^2(\Omega )}=1\right\} . \end{aligned}$$

We assert that $\lambda _1>0$ is the smallest eigenvalue associated to $(-\Delta )^s$. To see this, let $(u_k)_{k\in {{\mathbb {N}}}}\subset \widetilde{H}^s(\Omega )$ be a minimizing sequence, that is

$$ \left\| u_k\right\| _{L^2(\Omega )}=1\text { and }\lim _{k\rightarrow \infty }\left\| u_k \right\| _{\widetilde{H}^s(\Omega )}^2=\lambda _1. $$

In particular, this implies that $\left( u_k\right) _{k\in {{\mathbb {N}}}}\subset \widetilde{H}^s(\Omega )$ is uniformly bounded and hence up to extracting a subsequence there exists $\phi _1\in \widetilde{H}^s(\Omega )$ such that $u_k\rightharpoonup \phi _1$ in $\widetilde{H}^s(\Omega )$ as $k\rightarrow \infty$. Up to extraction of a further subsequence, we can assume by the Rellich-Kondrachov theorem that $u_k\rightarrow \phi _1$ in $\widetilde{L}^2(\Omega )$ as $k\rightarrow \infty$ (we still denote the subsequence by $\left( u_k\right) _{k\in {{\mathbb {N}}}}$). The latter condition guarantees $\left\| \phi _1\right\| _{L^2(\Omega )}=1$. Additionally, the lower semicontinuity of weak convergence ensures that $\Vert \phi _1\Vert _{\widetilde{H}^s(\Omega )}^2=\lambda _1$. Thus, $\phi _1\in \widetilde{H}^s(\Omega )$ is a minimizer of the convex functional $u\mapsto \Vert u\Vert _{\widetilde{H}^s(\Omega )}^2$, whose Euler–Lagrange equation is

$$ \big \langle (-\Delta )^{s/2}\phi _1,(-\Delta )^{s/2}v \big \rangle _{L^2({{\mathbb {R}}}^n)}=\lambda _1\left\langle \phi _1,v \right\rangle _{L^2({{\mathbb {R}}}^n)} $$

for all $v\in \widetilde{H}^s(\Omega )$ (see [30, Theorem 2.1]). This is nothing else than that $\lambda _1>0$ is an eigenvalue and $\phi _1\in \widetilde{H}^s(\Omega )$ is a related eigenfunction. That is, $\phi _1$ solves

$$ {\left\{ \begin{array}{ll} (-\Delta )^su =\lambda _1 u & \text { in }\Omega ,\\ u=0 & \text { in }\Omega _e. \end{array}\right. } $$

Next, we show that $\lambda _1>0$ is the smallest eigenvalue. For this purpose, assume that $\lambda >0$ is any eigenvalue with normalized eigenfunction $\psi \in \widetilde{H}^s(\Omega )$. Then we have

$$ \Vert \psi \Vert _{L^2(\Omega )}=1\text { and } \Vert \psi \Vert _{\widetilde{H}^s(\Omega )}^2=\lambda . $$

By the definition of $\lambda _1$, we get $\lambda \ge \lambda _1$.

*Step 2. The* *k*-*th eigenvalue*.

Let $k\ge 2$. Then we define

A.1 $$\begin{aligned} \begin{aligned} \lambda _k =&\inf \Big \{\Vert u\Vert _{\widetilde{H}^s(\Omega )}^2\,;\,u\in \widetilde{H}^s(\Omega ),\,\Vert u\Vert _{L^2(\Omega )}=1,\\&\qquad \quad \quad \quad \quad \quad \, \left\langle u,\phi _{\ell }\right\rangle _{L^2(\Omega )}=0\text { for }1\le \ell \le k-1\Big \}, \end{aligned} \end{aligned}$$

where $\phi _1,\ldots ,\phi _{k-1}$ are the normalized eigenfunctions corresponding to the eigenvalues $\lambda _1,\ldots ,\lambda _{k-1}$ and for all $1\le \ell \le k-1$ we have

$$\begin{aligned} \left\langle \phi _{\ell },\phi _m\right\rangle _{L^2(\Omega )}=0\text { for }1\le m\le \ell -1. \end{aligned}$$

Let us assume that that statement holds for $k-1$ and we aim to prove that it holds for *k*. As above, we take a minimizing sequence $\left( u_\ell \right) _{\ell \in {{\mathbb {N}}}}$ of (A.1) and, up to subtracting a subsequence, we can assume that

$$ u_\ell \rightharpoonup \phi _k \quad \text {in}\quad \widetilde{H}^s(\Omega )\quad \text {and}\quad u_\ell \rightarrow \phi _k\quad \text {in}\quad \widetilde{L}^2(\Omega ) $$

as $\ell \rightarrow \infty$ for some $\phi _k\in \widetilde{H}^s(\Omega )$. Furthermore, one can easily see that there holds

A.2 $$\begin{aligned} \left\| \phi _k\right\| _{L^2(\Omega )}=1,\, \left\langle \phi _k,\phi _\ell \right\rangle _{L^2(\Omega )}=0\text { for }1\le \ell \le k-1\text { and } \lambda _k=\left\| \phi _k\right\| _{\widetilde{H}^s(\Omega )}^2. \end{aligned}$$

Thus, $\phi _k$ is a minimizer with $\left\| \phi _k\right\| _{\widetilde{H}^s(\Omega )}^2=\lambda _k$. Next, let us define

$$ \begin{aligned} w_t{:}= \phi _k+tv-\sum _{\ell =1}^{k-1}\left\langle \phi _k+tv,\phi _{\ell }\right\rangle _{L^2(\Omega )}\phi _\ell =\phi _k+t\bigg (v-\sum _{\ell =1}^{k-1}\left\langle v,\phi _{\ell }\right\rangle _{L^2(\Omega )}\phi _{\ell }\bigg ) \end{aligned} $$

for $t\in {{\mathbb {R}}}$ and $v\in \widetilde{H}^s(\Omega )\setminus \{0\}$. Thus, we may estimate

$$ \begin{aligned} \left\| w_t\right\| _{L^2(\Omega )}&\ge \left\| \phi _k \right\| _{L^2(\Omega )}-|t|\bigg \Vert v-\sum _{\ell =1}^{k-1}\left\langle v,\phi _{\ell }\right\rangle _{L^2(\Omega )}\phi _{\ell }\bigg \Vert _{L^2(\Omega )}\\&\ge 1-|t|\bigg ( \Vert v\Vert _{L^2(\Omega )}+\sum _{\ell =1}^{k-1}\big |\left\langle v,\phi _\ell \right\rangle _{L^2(\Omega )}\big |\Vert \phi _\ell \Vert _{L^2(\Omega )}\bigg )\\&\ge 1-k|t|\Vert v\Vert _{L^2(\Omega )}>0 \end{aligned} $$

as long as $|t|<\frac{1}{k}\Vert v\Vert _{L^2(\Omega )}$, where we used that $\Vert \phi _\ell \Vert _{L^2(\Omega )}=1$ for $1\le \ell \le k$. Therefore, we can define

$$ \widetilde{w}_t=\frac{w_t}{\left\| w_t\right\| _{L^2(\Omega )}} $$

for $|t|<\frac{1}{k}\Vert v\Vert _{L^2(\Omega )}$. Using (A.2) and setting $\widetilde{v}=v-\sum _{\ell =1}^{k-1}\left\langle v,\phi _{\ell }\right\rangle _{L^2(\Omega )}\phi _{\ell }$, we deduce that there holds

$$ \left. \frac{d}{dt}\right| _{t=0}\left\| w_t\right\| _{L^2(\Omega )}^2=2\langle \phi _k,\widetilde{v}\rangle _{L^2(\Omega )}=2 \left\langle \phi _k,v \right\rangle _{L^2(\Omega )}, $$

and

$$ \begin{aligned} 0&=\left. \frac{d}{dt}\right| _{t=0}\left\| \widetilde{w}_t\right\| _{\widetilde{H}^s(\Omega )}^2\\&=\left. \frac{d}{dt}\right| _{t=0}\frac{\left\| w_t\right\| _{\widetilde{H}^s(\Omega )}^2}{\left\| w_t\right\| _{L^2(\Omega )}^2}\\&=\left. \frac{d}{dt}\right| _{t=0} \left\| w_t\right\| _{\widetilde{H}^s(\Omega )}^2- \lambda _k\left. \frac{d}{dt}\right| _{t=0}\left\| w_t\right\| _{L^2(\Omega )}^2\\&=2\big (\big \langle (-\Delta )^{s/2}\phi _k,(-\Delta )^{s/2}\widetilde{v}\big \rangle _{L^2({{\mathbb {R}}}^n)}-\lambda _k\left\langle \phi _k,v\right\rangle _{L^2(\Omega )}\big )\\&=2\big (\big \langle (-\Delta )^{s/2}\phi _k,(-\Delta )^{s/2}v\big \rangle _{L^2({{\mathbb {R}}}^n)}-\lambda _k\left\langle \phi _k,v\right\rangle _{L^2(\Omega )}\big ). \end{aligned} $$

In the last equality, we used that $\phi _\ell$ for $1\le \ell \le k-1$ are eigenfunctions of the fractional Laplacian and in $\widetilde{L}^2(\Omega )$ orthogonal to $\phi _k$ by (A.2). The above computation shows that $\lambda _k$ is an eigenvalue and $\phi _k$ a corresponding eigenfunction.

Next, we assert that $\lambda _k\rightarrow \infty$ as $k\rightarrow \infty$. Suppose for the sake of contradiction that $\lambda _k$ is uniformly bounded, so that also $\left( \phi _k\right) _{k\in {{\mathbb {N}}}}\subset \widetilde{H}^s(\Omega )$ is uniformly bounded. Thus, up to extracting a subsequence, $\left( \phi _k\right) _{k\in {{\mathbb {N}}}}$ converges in $\widetilde{L}^2(\Omega )$ and in particular is a Cauchy sequence. But then by the above construction, we have $\left\| \phi _k-\phi _m\right\| _{L^2(\Omega )}^2=\left\| \phi _k\right\| _{L^2(\Omega )}^2 +\left\| \phi _m\right\| _{L^2(\Omega )}^2+2\left\langle \phi _k,\phi _m\right\rangle _{L^2(\Omega )}=2$, for $k\ne m$ and thus $\left( \phi _k\right) _{k\in {{\mathbb {N}}}}$ cannot be Cauchy, a contradiction. Therefore, we necessarily have $\lambda _k\rightarrow \infty$ as $k\rightarrow \infty$.

*Step 3. Proof of * (i).

We already know that $(\phi _k)_{k\in {{\mathbb {N}}}}$ is an orthonormal system in $\widetilde{L}^2(\Omega )$. So, we only need to establish that the linear span of $(\phi _k)_{k\in {{\mathbb {N}}}}$ is dense in $\widetilde{L}^2(\Omega )$. As $\widetilde{H}^s(\Omega )$ is dense in $\widetilde{L}^2(\Omega )$ and $\left( \phi _k\right) _{k\in {{\mathbb {N}}}}\subset \widetilde{H}^s(\Omega )$, it is enough to show that every function in $\widetilde{H}^s(\Omega )$ can be approximated by elements in the linear span of $(\phi _k)_{k\in {{\mathbb {N}}}}$. So, let $v\in \widetilde{H}^s(\Omega )$ be any fixed function and define

A.3 $$\begin{aligned} v_k=v-\sum _{\ell =1}^{k-1}\left\langle v,\phi _{\ell }\right\rangle _{L^2(\Omega )}\phi _\ell =v-\sum _{\ell =1}^{k-1}\lambda _\ell ^{-1}\left\langle v,\phi _{\ell }\right\rangle _{\widetilde{H}^s(\Omega )}\phi _\ell \end{aligned}$$

for any $k\ge 2$. By orthonormality of $(\phi _k)_{k\in {{\mathbb {N}}}}$ in $\widetilde{L}^2(\Omega )$, we get $\left\langle v_k,\phi _\ell \right\rangle _{L^2(\Omega )}=0\text { for any }1\le \ell \le k-1$. By formula (A.1) this yields

A.4 $$\begin{aligned} \left\| v_k\right\| _{\widetilde{H}^s(\Omega )}^2\ge \lambda _k \left\| v_k\right\| _{L^2(\Omega )}^2. \end{aligned}$$

Now, using the orthonormality of $\left( \phi _\ell \right) _{1\le \ell \le k-1}$, we may compute

A.5 $$\begin{aligned} \begin{aligned} \Vert v\Vert _{\widetilde{H}^s(\Omega )}^2&=\bigg \Vert v_k+\sum _{\ell =1}^{k-1}\lambda _{\ell }^{-1}\langle v,\phi _\ell \rangle _{\widetilde{H}^s(\Omega )}\phi _\ell \bigg \Vert ^2_{\widetilde{H}^s(\Omega )}\\&=\left\| v_k\right\| _{\widetilde{H}^s(\Omega )}^2+2\sum _{\ell =1}^{k-1}\lambda _{\ell }^{-1}\left\langle v,\phi _\ell \right\rangle _{\widetilde{H}^s(\Omega )}\langle v_k,\phi _\ell \rangle _{\widetilde{H}^s(\Omega )}\\&\quad \, +\sum _{\ell =1}^{k-1}\sum _{\ell '=1}^{k-1}\lambda _{\ell }^{-1}\lambda _{\ell '}^{-1}\left\langle v,\phi _\ell \right\rangle _{\widetilde{H}^s(\Omega )}\left\langle v,\phi _{\ell '}\right\rangle _{\widetilde{H}^s(\Omega )}\left\langle \phi _{\ell },\phi _{\ell '}\right\rangle _{\widetilde{H}^s(\Omega )}\\&=\left\| v_k\right\| _{\widetilde{H}^s(\Omega )}^2+\sum _{\ell =1}^{k-1}\lambda _{\ell }^{-1}\big |\left\langle v,\phi _{\ell }\right\rangle _{\widetilde{H}^s(\Omega )}\big |^2\\&\ge \left\| v_k\right\| _{\widetilde{H}^s(\Omega )}^2. \end{aligned} \end{aligned}$$

Thus, by (A.4) we obtain

$$ \left\| v_k\right\| _{L^2(\Omega )}^2 \le \lambda _k^{-1}\left\| v_k\right\| _{\widetilde{H}^s(\Omega )}^2\le \lambda _k^{-1}\Vert v\Vert _{\widetilde{H}^s(\Omega )}^2. $$

Passing to the limit, this implies $v_k\rightarrow 0$ in $\widetilde{L}^2(\Omega )$. Hence, we have

$$\begin{aligned} v=\sum _{\ell =1}^{\infty }\left\langle v,\phi _{\ell }\right\rangle _{L^2(\Omega )}\phi _\ell =\sum _{\ell =1}^{\infty }\big \langle v,\lambda _{\ell }^{-1/2}\phi _{\ell }\big \rangle _{\widetilde{H}^s(\Omega )}\big (\lambda _{\ell }^{-1/2} \phi _\ell \big ) \end{aligned}$$

in $$\widetilde{L}^2(\Omega )$$.

*Step 4. Proof of * (ii).

First note that $\big ( \lambda _k^{-1/2}\phi _k\big )_{k\in {{\mathbb {N}}}}\subset \widetilde{H}^s(\Omega )$ is orthonormal. This is a direct consequence of the above construction. It remains to show the density of the linear span of $\big ( \lambda _k^{-1/2}\phi _k\big )_{k\in {{\mathbb {N}}}}$ in $\widetilde{H}^s(\Omega )$. Let us fix $v\in \widetilde{H}^s(\Omega )$ and suppose that the sequence $\left( v_k\right) _{k\ge 2}$ is defined as in (A.3). From the estimate (A.5) we know that $\left( v_k\right) _{k\ge 2}$ is uniformly bounded in $\widetilde{H}^s(\Omega )$ and thus up to extracting a subsequence, we get $v_k\rightharpoonup w$ in $\widetilde{H}^s(\Omega )$ for some $w\in \widetilde{H}^s(\Omega )$. The compact embedding $\widetilde{H}^s(\Omega )\hookrightarrow \widetilde{L}^2(\Omega )$ now gives $w=0$ as we already know from the previous step that $v_k\rightarrow 0$ in $\widetilde{L}^2(\Omega )$ as $k\rightarrow \infty$. As for any subsequence, there is a further subsequence with this property, we know that the whole sequence weakly converges in $\widetilde{H}^s(\Omega )$ to this limit $w=0$. By Mazur’s lemma, there exists a sequence of convex linear combinations

$$ w_\ell =\sum _{k=2}^{\ell }a_{k}^{(\ell )}v_k,\quad 0\le a_k^{(\ell )}\le 1,\quad \sum _{k=2}^{\ell }a_{k}^{(\ell )}=1 $$

such that $w_\ell \rightarrow 0$ in $\widetilde{H}^s(\Omega )$. Note that by (A.3) we have

$$ w_\ell =v-\sum _{k=2}^{\ell }\sum _{m=1}^{k-1}a_k^{(\ell )}\big \langle v,\lambda _m^{-1/2}\phi _m \big \rangle _{\widetilde{H}^s(\Omega )}\big (\lambda _m^{-1/2}\phi _m\big ) $$

and thus

$$ W_\ell =\sum _{k=2}^{\ell }\sum _{m=1}^{k-1}a_k^{(\ell )}\big \langle v,\lambda _m^{-1/2}\phi _m \big \rangle _{\widetilde{H}^s(\Omega )}\big (\lambda _m^{-1/2}\phi _m\big )\rightarrow v $$

in $\widetilde{H}^s(\Omega )$ as $\ell \rightarrow \infty$. As the functions $W_\ell$ clearly belong to the span of $\big (\lambda _k^{-1/2}\phi _k\big )_{k\in {{\mathbb {N}}}}$, we may conclude the proof.

*Step 5. Proof of * (iii).

Note that for any $G\in H^{-s}(\Omega )$ and $v\in \widetilde{H}^s(\Omega )$ we have by (ii) the identity

$$ \langle G,v\rangle =\sum _{k=1}^{\infty }\big \langle v,\lambda _k^{-1/2}\phi _k \big \rangle _{\widetilde{H}^s(\Omega )} \big \langle G,\lambda _k^{-1/2}\phi _k\big \rangle . $$

Using the Cauchy-Schwarz inequality, we get

$$ \begin{aligned} |\langle G,v\rangle |&\le \sum _{k=1}^{\infty }\big |\big \langle v,\lambda _k^{-1/2}\phi _k\big \rangle _{\widetilde{H}^s(\Omega )}\big |\lambda _k^{-1/2}\big |G_k\big |\\&\le \bigg (\sum _{k=1}^{\infty }\big |\big \langle v,\lambda _k^{-1/2}\phi _k\big \rangle _{\widetilde{H}^s(\Omega )} \big |^2\bigg )^{1/2}\bigg (\sum _{k=1}^{\infty }\lambda _k^{-1}\left| G_k\right| ^2\bigg )^{1/2}\\&= \Vert v\Vert _{\widetilde{H}^s(\Omega )}\bigg (\sum _{k=1}^{\infty }\lambda _k^{-1}\left| G_k\right| ^2\bigg )^{1/2}, \end{aligned} $$

where we have again put $G_k=\langle G,\phi _k\rangle$ and used [7, Corollary 5.10]. Hence,

A.6 $$\begin{aligned} \Vert G\Vert _{H^{-s}(\Omega )}\le \bigg (\sum _{k=1}^{\infty }\lambda _k^{-1}|G_k|^2\bigg )^{1/2}. \end{aligned}$$

Next, let $v\in \widetilde{H}^s(\Omega )$ be the unique solution to

A.7 $$\begin{aligned} {\left\{ \begin{array}{ll} (-\Delta )^s v=G & \text { in }\Omega ,\\ v=0 & \text { in }\Omega _e, \end{array}\right. } \end{aligned}$$

which exists by the Lax-Milgram theorem, and satisfies

A.8 $$\begin{aligned} \Vert v\Vert _{\widetilde{H}^s(\Omega )}\le \Vert G\Vert _{H^{-s}(\Omega )}. \end{aligned}$$

By Plancherel’s theorem, the left-hand side can be written as

A.9 $$\begin{aligned} \Vert v\Vert _{\widetilde{H}^s(\Omega )}^2=\sum _{k=1}^{\infty }\big |\big \langle v,\lambda _k^{-1/2}\phi _k\big \rangle _{\widetilde{H}^s(\Omega )}\big |^2. \end{aligned}$$

As *v* solves (A.7), we get

A.10 $$\begin{aligned} \begin{aligned} \big \langle v,\lambda _k^{-1/2}\phi _k\big \rangle _{\widetilde{H}^s(\Omega )}&=\lambda _k^{-1/2}\big \langle (-\Delta )^{s/2}v,(-\Delta )^{s/2}\phi _k\big \rangle _{L^2({{\mathbb {R}}}^n)}\\&=\lambda _k^{-1/2}\left\langle G,\phi _k\right\rangle \\&=\lambda _k^{-1/2}G_k. \end{aligned} \end{aligned}$$

Taking into account (A.8) and (A.9), we get

A.11 $$\begin{aligned} \begin{aligned} \bigg (\sum _{k=1}^{\infty }\lambda _k^{-1}\left| G_k\right| ^2\bigg )^{1/2}&= \bigg (\sum _{k=1}^{\infty }\big |\big \langle v,\lambda _k^{-1/2}\phi _k\big \rangle _{\widetilde{H}^s(\Omega )}\big |^2\bigg )^{1/2}\\&=\Vert v\Vert _{\widetilde{H}^s(\Omega )}\\&\le \Vert G\Vert _{H^{-s}(\Omega )}. \end{aligned} \end{aligned}$$

Thus, we may conclude that for any $G\in H^{-s}(\Omega )$, we have

A.12 $$\begin{aligned} \Vert G\Vert _{H^{-s}(\Omega )}=\bigg (\sum _{k=1}^{\infty }\lambda _k^{-1}\left| G_k\right| ^2\bigg )^{1/2}. \end{aligned}$$

Next, we assert that for any $G\in H^{-s}(\Omega )$ we have

A.13 $$\begin{aligned} G=\sum _{k=1}^{\infty }G_k\phi _k\quad \text {in}\quad H^{-s}(\Omega ), \end{aligned}$$

where $G_k=\left\langle G,\phi _k \right\rangle$, for $k\in {{\mathbb {N}}}$. Again, let $v\in \widetilde{H}^s(\Omega )$ be the unique solution of (A.7). Then from (A.6) and (A.11), we know that

A.14 $$\begin{aligned} \Vert v\Vert _{\widetilde{H}^s(\Omega )}=\Vert G\Vert _{H^{-s}(\Omega )}. \end{aligned}$$

Therefore the source-to-solution map $S:H^{-s}(\Omega )\rightarrow \widetilde{H}^s(\Omega )$ related to (A.7) is an isometric isomorphism. In fact, surjectivity follows by using $G=(-\Delta )^s v\in H^{-s}({{\mathbb {R}}}^n)\hookrightarrow H^{-s}(\Omega )$ for given $v\in \widetilde{H}^s(\Omega )$ as a source. By (ii), we already know that

$$ v=\sum _{k=1}^{\infty }\big \langle v,\lambda _k^{-1/2}\phi _k\big \rangle _{\widetilde{H}^s(\Omega )}\lambda _k^{-1/2}\phi _k $$

in $\widetilde{H}^s(\Omega )$ for any $v\in \widetilde{H}^s(\Omega )$. As $SG=v$ and $S^{-1}$ is a bounded linear map by the Banach isomorphism theorem, we deduce that

$$ G=S^{-1}v=\sum _{k=1}^{\infty }\lambda _k^{-1/2}\big \langle v,\lambda _k^{-1/2}\phi _k\big \rangle _{\widetilde{H}^s(\Omega )}S^{-1}\phi _k\quad \text {in}\quad H^{-s}(\Omega ). $$

As $S^{-1}\phi _k=\lambda _k\phi _k$, we get by (A.10) the identity

A.15 $$\begin{aligned} \begin{aligned} G&=\sum _{k=1}^{\infty }\lambda _k^{1/2}\big \langle v,\lambda _k^{-1/2}\phi _k\big \rangle _{\widetilde{H}^s(\Omega )}\phi _k\\&=\sum _{k=1}^{\infty }G_k\phi _k\quad \text {in}\quad H^{-s}(\Omega ). \end{aligned} \end{aligned}$$

This verifies the assertion (A.13). Observe that the bilinear form

A.16 $$\begin{aligned} \langle G,H\rangle _{H^{-s}(\Omega )}{:}=\langle SG,SH\rangle _{\widetilde{H}^s(\Omega )} \end{aligned}$$

for $G,H\in H^{-s}(\Omega )$ defines an inner product on $H^{-s}(\Omega )$ and the induced norm coincides with the dual norm $\Vert \cdot \Vert _{H^{-s}(\Omega )}$ (see (A.14)). Note that

$$ \begin{aligned} \left\langle \phi _k,\phi _\ell \right\rangle _{H^{-s}(\Omega )}&=\left\langle S\phi _k,S\phi _{\ell }\right\rangle _{\widetilde{H}^s(\Omega )}=\lambda _k^{-1}\lambda _{\ell }^{-1}\left\langle \phi _k,\phi _{\ell }\right\rangle _{\widetilde{H}^s(\Omega )}\\&=\lambda _{\ell }^{-1}\left\langle \phi _k,\phi _{\ell }\right\rangle _{L^2(\Omega )}=\lambda _k^{-1}\delta _{k,\ell } \end{aligned} $$

for any $k,\ell \in {{\mathbb {N}}}$. Hence, $\big ( \lambda _k^{1/2}\phi _k\big )_{k\in {{\mathbb {N}}}}$ is orthonormal in $H^{-s}(\Omega )$. By the definition of the isomorphism *S*, $S\phi _k=\lambda _k^{-1}\phi _k$ and (A.16), we get

A.17 $$\begin{aligned} \begin{aligned} G_k&=\langle G,\phi _k\rangle \\&=\left\langle SG,\phi _k\right\rangle _{\widetilde{H}^s(\Omega )}\\&=\left\langle SG,S (\lambda _k\phi _k)\right\rangle _{\widetilde{H}^s(\Omega )}\\&=\lambda _k\left\langle G,\phi _k\right\rangle _{H^{-s}(\Omega )}. \end{aligned} \end{aligned}$$

Finally, by (A.15) this implies

$$\begin{aligned} G=\sum _{k=1}^{\infty } \big \langle G,\lambda _k^{1/2}\phi _k\big \rangle _{H^{-s}(\Omega )}\lambda _k^{1/2}\phi _k\quad \text {in}\quad H^{-s}(\Omega ), \end{aligned}$$

which in turn implies that $\big (\lambda _k^{1/2}\phi _k\big )_{k\in {{\mathbb {N}}}}$ is an orthonormal basis in $H^{-s}(\Omega )$. $\square$

## Footnotes

1 This assumption is included for simplicity and the result remains true, for example, if one assumes instead $q\in L^{\infty }(\Omega )$.

2 Alternatively, one can use the well-known Moore–Osgood theorem together with (5.17).
